# Mitochondrial 8-Oxoguanine DNA Glycosylase 1–Mitochondrial Permeability Transition Pore Axis Drives Mitochondrial DNA Escape and Accelerates Osteoarthritis Progression

**DOI:** 10.34133/research.1235

**Published:** 2026-04-15

**Authors:** Shiqian Huang, Heting Yu, Weizhong Qi, Na Lin, Jianmao Chen, Hong Huang, Pengcheng Hu, Ziqi Zhou, Mengdi Zhang, Guangfeng Ruan, Song Xue, Changhai Ding

**Affiliations:** ^1^ Clinical Research Centre, Zhujiang Hospital, Southern Medical University, Guangzhou, China.; ^2^ Clinical Research Center, Guangzhou First People’s Hospital, Guangzhou Medical University, Guangzhou, China.; ^3^ Department of Sports Medicine and Rehabilitation, Peking University Shenzhen Hospital, Shenzhen Peking University–The Hong Kong University of Science and Technology Medical Center, Shenzhen, China.; ^4^ Menzies Institute for Medical Research, University of Tasmania, Hobart, Australia.; ^5^ Clinical Research Centre, Beijing Tsinghua Changgung Hospital, Tsinghua Medicine, Tsinghua University, Beijing, China.

## Abstract

Mitochondrial DNA (mtDNA) damage and its subsequent release into the cytoplasm are strongly linked to osteoarthritis (OA), but the pathogenic mechanism remains poorly understood. Here, this study reveals that under inflammatory or oxidative stress, the down-regulation of mitochondrial base excision repair enzyme 8-oxoguanine DNA glycosylase 1 and excessive opening of the mitochondrial permeability transition pore jointly drive mtDNA escape into the cytoplasm. Activation of 8-oxoguanine DNA glycosylase 1 with TH10785 reduces the production of oxidized mtDNA and preserves mtDNA integrity, while suppression of excessive mitochondrial permeability transition pore opening with cyclosporin A prevents mtDNA translocation. The combined intervention synergistically decreases cytosolic mtDNA levels, alleviating cartilage matrix degradation and cellular senescence. Mechanistically, cytosolic mtDNA induces the senescence-associated secretory phenotype by activating the cyclic guanosine monophosphate-adenosine monophosphate synthase–stimulator of interferon genes–nuclear factor κB signaling axis, whereas combined intervention blocks this cascade activation. Notably, intra-articular injection of the combination of TH10785 and cyclosporin A markedly reduces senescence and ameliorates the progression of the experimental OA model mice. This research reveals the dual regulatory roles of mtDNA integrity and translocation in governing cytosolic mtDNA content, providing novel insights for developing mtDNA-targeted therapeutic strategies against OA.

## Introduction

As a highly prevalent musculoskeletal disorder among the elderly, osteoarthritis (OA) is featured by persistent aseptic inflammation and gradual degradation of articular cartilage [1]. As a key subcellular organelle within cells, mitochondrial dysfunction (e.g., accumulation of damaged mitochondria and impairment of mitochondrial nucleic acid integrity) is strongly correlated with the initiation and advancement of OA [2]. Although the pathogenic mechanism of OA remains unclear, the pathological efflux of mitochondrial DNA (mtDNA) into the cytoplasm potentially represents a critical link connecting oxidative stress, inflammatory response, and cartilage degradation [3–5]. A previous study has shown that oxidative stress induces oxidative damage and fragmentation of mtDNA through excessive formation of mitochondrial reactive oxygen species (mtROS), thereby triggering the abnormal efflux of oxidized mtDNA (ox-mtDNA) from mitochondria to the cytoplasm and activating cellular inflammatory cascades [6]. However, the specific mechanisms governing the dynamic regulation of cytosolic mtDNA in OA have yet to be fully clarified, and the lack of effective interventions targeting this process poses a significant challenge for OA therapy.

The restoration of mtDNA oxidative damage, a process pivotal to modulating cytosolic mtDNA content, involves a precisely regulated balance between mtDNA repair and degradation systems to maintain mtDNA stability [7–9]. Of note, the enzymes that regulate the mtDNA repair or degradation systems can reduce the production of cytosolic mtDNA in chronic inflammation [4,6,10]. Among them, the key mitochondrial base excision repair enzyme 8-oxoguanine DNA glycosylase 1 (OGG1) can recognize and excise 8-hydroxydeoxyguanosine (8OHdG) generated by oxidative stress in mtDNA, thereby preventing excessive formation of ox-mtDNA and mitochondrial dysfunction [11,12]. Recent research has revealed that enhancing mitochondrial OGG1 activity specifically reduces the efflux of ox-mtDNA into the cytoplasm in an oxidative-stress-dominated inflammatory microenvironment, suggesting that it directly regulates cytosolic mtDNA content through oxidative damage repair mechanisms [6,13,14]. However, it remains undefined whether OGG1 is implicated in the dynamic regulation of cytosolic ox-mtDNA within the context of OA.

Besides, disruption of mitochondrial membrane homeostasis serves as a direct inducer of mtDNA cytosolic release under oxidative stress [15,16]. Elevated oxidative stress is responsible for initiating the opening of the mitochondrial permeability transition pore (mPTP) through increased calcium influx and mitochondrial membrane potential (Δ*Ψm*) depletion [17–19]. mPTP opening contributes to transient receptor potential vanilloid 4-mediated mitochondrial impairment in chondrocytes [20]. In addition, mPTP opening is critical for mtDNA to breach subcellular barriers and release into the cytoplasm [16,21]. However, the dynamic relationship between mtDNA oxidative damage repair and mPTP opening remains undefined, with potential temporal regulation or synergistic mechanisms governing their interplay in mtDNA cytosolic escape.

Senescence is prevalent in degenerative and proliferative age-related diseases, characterized by augmented senescence-associated β-galactosidase (SA-β-gal) activity, shortened telomere length, and accumulation of DNA damage [22,23]. Among these, mtDNA mutations and oxidative stress are recognized as key contributors to chondrocyte senescence in OA [24–26]. Previous studies have demonstrated that excessively accumulated cytosolic DNA, acting as damage-associated molecular patterns (DAMPs), is detected by pattern recognition receptors, thereby initiating innate immune responses and ultimately promoting the senescence-associated secretory phenotype (SASP) [27–30]. Among them, the cyclic guanosine monophosphate–adenosine monophosphate (cGAMP) synthase-stimulator of interferon genes (cGAS-STING) pathway, a key branch of the innate immune inflammatory response, has been increasingly validated as a sensor of cytosolic mtDNA [31,32]. A recent study showed that Piezo1-mediated mtDNA release triggers activation of the cGAS-STING pathway, thereby promoting the release of proinflammatory cytokines in chondrocytes [4]. However, the relationship between cytosolic mtDNA-mediated immune-inflammatory pathways and cartilage senescence during OA progression is still unknown.

In our study, we hypothesize that mitochondrial-dysfunction-induced down-regulation of OGG1 activity and excessive mPTP opening may promote mtDNA release upon inflammatory or oxidative stress, thereby exacerbating cGAS-STING pathway-mediated tissue senescence. This research is designed to elucidate the roles of OGG1 activation and mPTP blockade in regulating cytosolic mtDNA content, cGAS-STING pathway activation, and senescence process. Exploring these mechanisms may provide some novel therapeutic targets for OA prevention and treatment.

## Results

### Abnormal cytosolic mtDNA release mediated chondrocyte degeneration

**Fig. 1.**
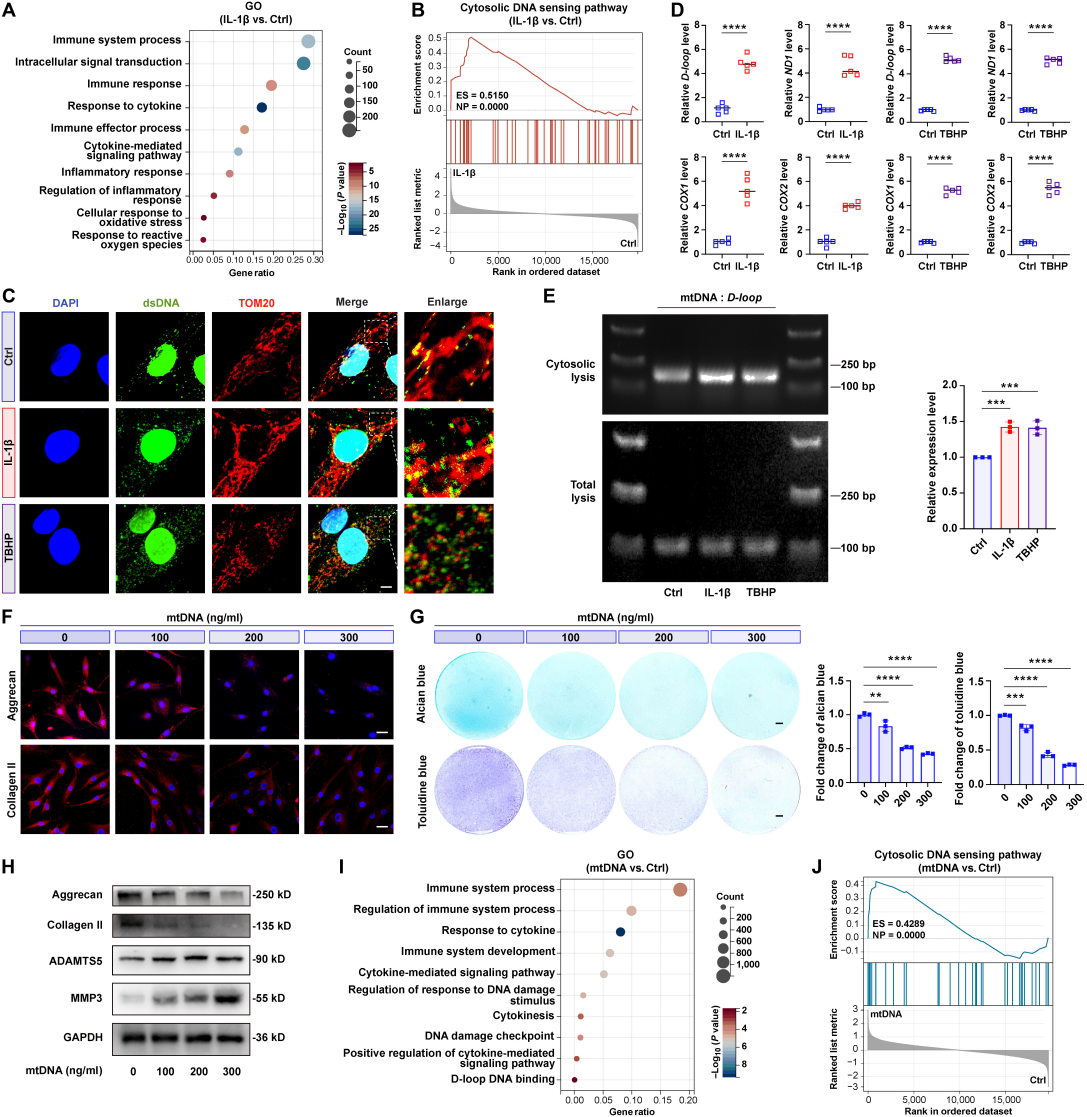
Oxidative and inflammatory stress triggered fragmented mitochondrial DNA (mtDNA) release into the cytoplasm and accelerated extracellular matrix (ECM) degradation in chondrocytes. (A) Gene Ontology (GO) analysis of differentially expressed genes showing enhanced immunity-associated pathways in interleukin-1β (IL-1β)-treated versus control human primary chondrocytes (HCs) (*n* = 3). (B) Gene set enrichment analysis (GSEA) of IL-1β versus control groups (*n* = 3) with enrichment in “cytosolic DNA sensing pathway”. ES, enrichment score; NP, normalized *P* value. (C) Representative images of double-stranded DNA (dsDNA) (green), translocase of outer mitochondrial membrane 20 (TOM20) (red), and 4′,6-diamidino-2-phenylindole (DAPI) (blue) in treated HCs. Scale bar, 10 μm. (D) Relative cytosolic mtDNA levels (*D-loop*, *ND1*, *COX1*, and *COX2*) in IL-1β- or *tert*-butyl hydroperoxide (TBHP)-treated HCs (*n* = 5). (E) DNA agarose electrophoresis images of cytosolic/total D-loop mtDNA in treated HCs (*n* = 3). (F) Representative images of aggrecan and collagen II in mtDNA-stimulated HCs (*n* = 3). Scale bars, 25 μm. (G) Staining and quantitative analysis of alcian blue and toluidine blue in mtDNA-treated HCs (*n* = 3). Scale bars, 5 μm. (H) Western blotting results of aggrecan, collagen II, a disintegrin and metalloproteinase with thrombospondin motifs 5 (ADAMTS5), and matrix metalloproteinase-3 (MMP3) expression in mtDNA-treated HCs (*n* = 3). (I) GO analysis of differentially expressed genes in mtDNA versus control groups (*n* = 3). (J) GSEA of mtDNA versus control groups (*n* = 3) with enrichment in “cytosolic DNA sensing pathway”. All bar graphs: means ± SD. ****P < 0.0001, ***P < 0.001, and **P < 0.01.

To investigate the underlying pathological mechanisms of OA chondrocyte dysfunction, we conducted RNA sequencing (RNA-seq) analysis on the affected chondrocytes. RNA-seq analysis results demonstrated a marked enrichment of differentially expressed genes in the inflammatory and immune-associated pathways, including immune system process, inflammatory response, etc. (Fig. 1A). Notably, gene set enrichment analysis (GSEA) results demonstrated that there was a marked activation of the “cytosolic DNA-sensing pathway” in affected chondrocytes (Fig. 1B). Previous research has established that abnormal mtDNA efflux into the cytoplasm serves as a potent driver of cascaded inflammatory responses, suggesting the potential involvement of cytosolic DNA in OA progression.

To assess mtDNA involvement in chondrocyte degradation pathogenesis, we then performed stimulation of inflammation and oxidative stress by treating human primary chondrocytes (HCs) with interleukin-1β (IL-1β; 10 ng/ml) or *tert*-butyl hydroperoxide (TBHP) (80 μM) for 24 h. These 2 stimuli represent the major pathological triggers in OA, with IL-1β recapitulating the inflammatory microenvironment and TBHP inducing oxidative stress, both of which contribute to chondrocyte degeneration. Both stimulations significantly promoted the reduction of anabolism-related proteins (aggrecan and collagen II) while up-regulating the catabolism-related proteins (a disintegrin and metalloproteinase with thrombospondin motifs 5 [ADAMTS5] and matrix metalloproteinase-3 [MMP3]) (Fig. S1A and B). Besides, both stimulations promoted extracellular matrix (ECM) degradation (Fig. S1C), indicating chondrocyte degradation establishment. Subsequently, fluorescence colocalization analysis of double-stranded DNA (dsDNA), mitochondria, and nuclei showed increased cytosolic dsDNA signals following either stimulation (Fig. 1C), indicating the elevated cytosolic DNA accumulation. This result was further corroborated by quantitative real-time polymerase chain reaction (qPCR) analysis of extracted cytosolic DNA. Specifically, the expression of mtDNA-encoded genes (*D-loop*, *ND1*, *COX1*, and *COX2*) was significantly elevated (Fig. 1D), providing direct evidence for mtDNA escape into the cytoplasm following both stimulations.

To delineate the pathological implications of cytosolic mtDNA in HCs dysfunction, we next analyzed the cytosolic mtDNA by agarose gel electrophoresis, which identified a distinct 100 to 250-bp fragmentation pattern in chondrocytes (Fig. 1E). Based on this, we further synthesized fragmented mtDNA in vitro and then stimulated HCs with the synthesized mtDNA at varying concentrations. As a result, the fragmented mtDNA induced the reduction of anabolism-related proteins and up-regulation of catabolism-related proteins in a concentration-dependent manner (Fig. 1F and H and Fig. S2A and B). Subsequently, alcian blue and toluidine blue staining results confirmed that the fragmented mtDNA accelerated ECM degradation (Fig. 1G). Furthermore, RNA-seq analysis results of mtDNA-stimulated HCs showed the enrichment of multiple immune-related pathways (Fig. 1I). In addition, mtDNA stimulation activated the cytosolic DNA-sensing pathway in HCs, corresponding to the effects observed in the IL-1β-induced chondrocytes (Fig. 1J). Collectively, these findings suggest that cytosolic mtDNA accumulation potentially contributes to OA pathogenesis.

### OGG1 down-regulation promoted ox-mtDNA accumulation and cytosolic release in chondrocytes

**Fig. 2.**
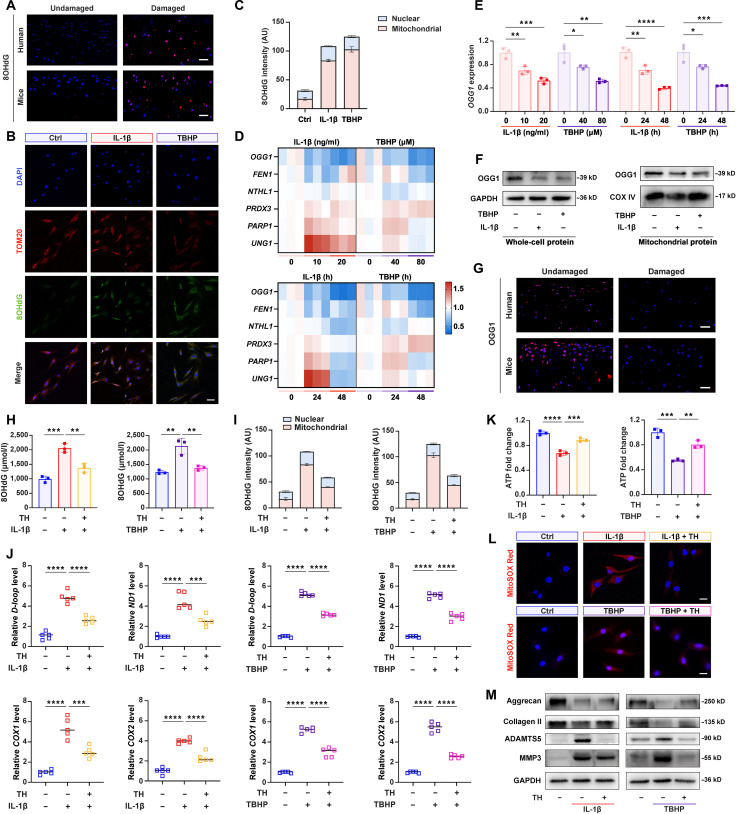
Down-regulation of 8-oxoguanine DNA glycosylase 1 (OGG1) promoted oxidized mitochondrial DNA (ox-mtDNA) generation and cytosolic release in chondrocytes, contributing to osteoarthritis (OA) pathogenesis. (A) Representative images of 8-hydroxydeoxyguanosine (8OHdG) (red) and DAPI (blue) in human and mouse OA cartilage and (B) 8OHdG (green), TOM20 (red), and DAPI (blue) in treated HCs (*n* = 3). Scale bars, 25 μm. (C) Relative 8OHdG intensity in the nuclear/mitochondria of (B). (D) Heatmap of reverse transcription quantitative real-time polymerase chain reaction (RT-qPCR) for DNA repair genes (*OGG1*, *FEN1*, etc.) and (E) RT-qPCR for OGG1 expression in HCs treated with IL-1β (0, 10, and 20 ng/ml) and TBHP (0, 40, and 80 μM) in a manner dependent on both dose and incubation time (0, 24, and 48 h) (*n* = 3). (F) Western blotting of OGG1 in the whole-cell/mitochondrial fractions of treated HCs (*n* = 3). (G) Representative images of OGG1 (red) and DAPI (blue) in human and mouse OA cartilage. Scale bars, 25 μm. (H) 8OHdG concentrations (*n* = 3), (I) relative 8OHdG intensity in nuclear/mitochondria (*n* = 3), (J) relative cytosolic mtDNA amounts (*n* = 5), (K) relative quantification of ATP production (*n* = 3), (L) representative MitoSOX Red images (scale bars, 20 μm) indicating mtROS (*n* = 3), and (M) western blotting results of aggrecan, collagen II, ADAMTS5, and matrix metalloproteinase-3 (MMP3) expression after TH (1 μM for 24 h) treatment (*n* = 3). All bar graphs: means ± SD. ****P < 0.0001, ***P < 0.001, **P < 0.01, and *P < 0.05.

Previous studies established that oxidative-damage-induced ox-mtDNA served as the primary trigger for mtDNA cytosolic release [6,33]. To evaluate oxidative damage in OA, we first quantified the DNA oxidation indicator 8OHdG in the cartilage. Immunohistochemical results revealed that there was a significant up-regulation of 8OHdG levels in both human OA cartilage and experimental mouse OA cartilage (Fig. 2A), demonstrating an up-regulation of oxidized DNA (ox-DNA) damage during OA progression. To precisely localize intracellular ox-DNA, we performed triple fluorescence colocalization analysis of 8OHdG with mitochondrial and nuclei. Quantitative analysis results demonstrated that there was a greater 8OHdG accumulation in mtDNA compared to nuclear DNA in both experimental stimulations (Fig. 2B and C), providing the definitive evidence that ox-mtDNA represented the predominant form of ox-DNA in affected chondrocytes.

Ox-mtDNA damage is likely driven by a disruption in the homeostatic balance between mtDNA repair activity and degradation dynamics [7,9]. To investigate the mechanisms underlying ox-mtDNA damage and cytosolic release, we focused on the key components of mtDNA repair system. Through systematic evaluation of genes involved in ox-mtDNA repair and degradation, we identified that the ox-mtDNA-specific repair enzyme OGG1 was gradually down-regulated in both a concentration-dependent and a time-dependent manner via reverse transcription qPCR (RT-qPCR) (Fig. 2D and E). The expression of OGG1 was also validated at the protein level. Both the whole-cell and mitochondrial OGG1 protein levels were significantly suppressed after stimulation with either IL-1β or TBHP for 24 h (Fig. 2F and Fig. S3A and B). Moreover, immunohistochemical analysis results consistently showed that there was a decreased expression of OGG1 both in human OA cartilage and destabilization of the medial meniscus (DMM)-induced mouse OA cartilage (Fig. 2G). These results demonstrate that the down-regulation of OGG1 may contribute to the accumulation of cytosolic ox-mtDNA by weakening mtDNA repair capacity.

To functionally characterize the role and mechanism of OGG1 in chondrocyte degeneration, we treated HCs with a specific OGG1 activator TH10785 (TH; 1 μM) for 24 h (Fig. S4) [34]. We found that TH treatment significantly suppressed the intracellular 8OHdG levels (Fig. 2H), demonstrating its efficacy in ameliorating ox-DNA damage. Subsequently, fluorescence colocalization and quantitative analysis results revealed that TH decreased ox-mtDNA generation within mitochondria (Fig. 2I and Fig. S5), indicating TH primarily activated the DNA repair function of mitochondrial OGG1. Furthermore, fluorescence colocalization results showed that TH treatment markedly attenuated IL-1β- or TBHP-induced cytosolic DNA accumulation (Fig. S6). In addition, there was a more significant reduction of cytosolic mtDNA from TH-treated HCs via qPCR analysis (Fig. 2J). These results demonstrate that OGG1 activation not only repairs mitochondrial ox-mtDNA damage but also effectively prevents its pathological leakage into the cytoplasm.

To evaluate the mitochondrial function of ox-mtDNA reduction, we assessed the energy metabolism status of chondrocytes following TH treatment for 24 h. The result revealed that TH administration not only enhanced adenosine triphosphate (ATP) production but also attenuated the mtROS generation upon both stimulations (Fig. 2K and L and Fig. S7A). Further assessment of the expression profile of metabolism-related proteins demonstrated that TH administration significantly up-regulated the expression of anabolism-related proteins (aggrecan and collagen II) while concurrently down-regulating catabolism-related proteins (ADAMTS5 and MMP3) (Fig. 2M and Fig. S7B).

Collectively, these observations indicate that the impaired OGG1 function promotes OA pathogenesis by facilitating both ox-mtDNA generation and its subsequent cytosolic release. TH-mediated OGG1 activation not only suppresses oxidative damage but also restores mitochondrial function and maintains chondrocyte metabolic balance.

### Blocking excessive mPTP opening restored the stabilization of Δ*Ψm*

**Fig. 3.**
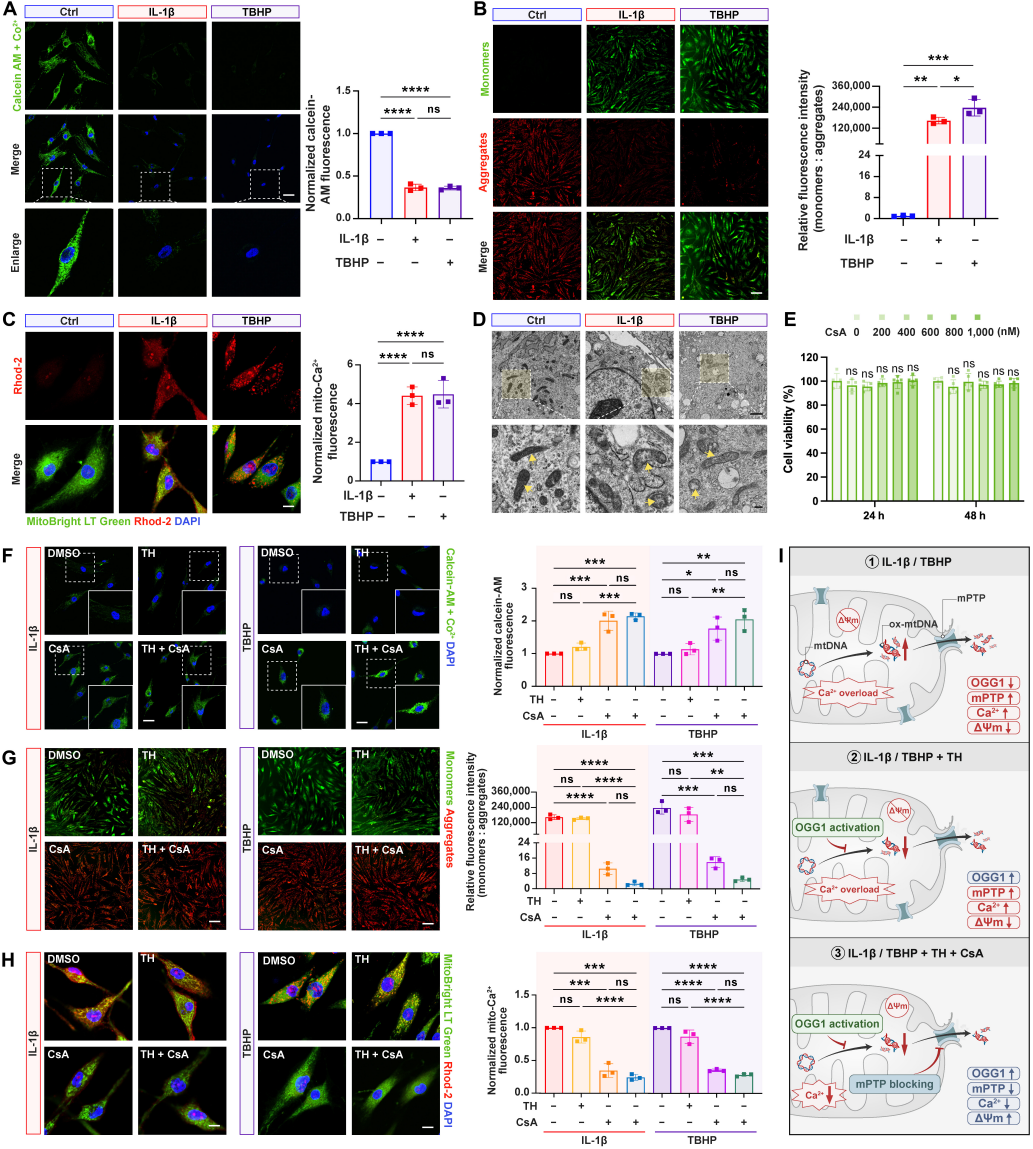
Inhibition of pathological mitochondrial permeability transition pore (mPTP) overactivation restored mitochondrial membrane homeostasis in stimulated chondrocytes. (A) Representative calcein-AM/Co^2+^ quencher staining fluorescence images, together with their quantification in treated HCs (*n* = 3). Scale bar, 25 μm. (B) Representative JC-1 staining images for mitochondrial membrane potential (Δ*Ψm*) alterations and the quantitative analysis in treated HCs (*n* = 3). Scale bar, 50 μm. (C) Representative Rhod-2/AM fluorescence images to indicate the mitochondrial calcium (mito-Ca^2+^) level and the quantification in the treated HCs (*n* = 3). Scale bar, 10 μm. (D) Representative transmission electron microscope (TEM) images of the treated chondrocytes. Yellow arrows indicate mitochondria. Scale bars, 1 μm (top) and 0.2 μm (bottom). (E) Effects of cyclosporin A (CsA) concentration gradient treatment on the proliferation capacity of HCs (*n* = 5). (F) Representative calcein-AM/Co^2+^ quencher staining fluorescence images with quantitative analysis, (G) representative JC-1 staining images, and (H) representative Rhod-2/AM fluorescent images in HCs after TH (1 μM for 24 h) or CsA (1 μM for 24 h) treatment (*n* = 3). Scale bars, 25 μm (F), 50 μm (G), and 10 μm (H). DMSO, dimethyl sulfoxide. (I) Schematic diagram illustrating that OGG1 activation alone cannot suppress mPTP opening but combined with CsA mitigates mitochondrial permeability transition. All bar graphs: means ± SD. ****P < 0.0001, ***P < 0.001, **P < 0.01, *P < 0.05, and *P* > 0.05 (not significant [ns]).

Although OGG1 has been confirmed to reduce cytosolic mtDNA release in affected chondrocytes, the underlying mechanisms of mtDNA extrusion remain unclear. Emerging evidence suggests that mtDNA release may result from the increased mitochondrial permeability, potentially mediated by the mPTP [17,18,35]. To clarify whether mPTP opening in IL-1β- or TBHP-treated HCs is triggered by inflammation or oxidative stress, we carried out fluorescence analysis. Our results showed that the calcein acetoxymethyl ester (calcein-AM) retention rate was significantly reduced in mitochondria after both stimulations (Fig. 3A), indicating substantial mPTP activation in the affected HCs. Considering that previous studies have revealed that mPTP opening decreases Δ*Ψm* while promoting mitochondrial calcium (mito-Ca^2+^) accumulation, we next conducted JC-1 staining to assess Δ*Ψm* and Rhod-2/AM positive staining to quantify mito-Ca^2+^ levels. JC-1 analysis revealed an increased monomer-to-aggregate ratio in the inflammation-challenged HCs (Fig. 3B), indicating Δ*Ψm* dissipation. Concurrently, colocalization of Rhod-2/AM and MitoTracker analysis results demonstrated that the mito-Ca^2+^ levels were elevated in the inflammation-challenged HCs (Fig. 3C), further supporting our hypothesis. The transmission electron microscope (TEM) results provided direct visual evidence of mitochondrial cristae disruption and matrix swelling in the inflammation-challenged HCs (Fig. 3D), collectively indicating that excessive mPTP opening destabilized mitochondrial membrane.

To investigate whether OGG1 activation improves the mitochondrial membrane permeability, we treated HCs with TH and further assessed mPTP opening degree. Intriguingly, although TH restored mitochondrial ATP production by reducing ox-mtDNA levels, it failed to significantly improve the calcein-AM retention in the inflammation-challenged HCs (Fig. 3E and F), indicating that OGG1 activation does not directly inhibit excessive mPTP opening. Notably, cotreatment with the mPTP inhibitor cyclosporin A (CsA; 1 μM) for 24 h markedly attenuated mPTP opening in the inflammation-challenged chondrocytes (Fig. 3E and F) [36]. Furthermore, the detection of Δ*Ψm* and mito-Ca^2+^ levels revealed that only CsA treatment effectively reversed both the increased JC-1 monomer/aggregate ratio (Fig. 3G) and mito-Ca^2+^ overload (Fig. 3H), whereas no significant alteration was observed in IL-1β-, TBHP-, or TH-treated groups.

These results collectively demonstrate that OGG1 activation alone is insufficient to suppress mPTP opening, whereas the combinatorial therapy with CsA effectively mitigates mitochondrial permeability transition and restores mitochondrial membrane homeostasis in HCs (Fig. 3I).

### Combination of OGG1 activation and mPTP blocking inhibited mtDNA cytosolic release and exerted chondroprotective effects in OA

**Fig. 4.**
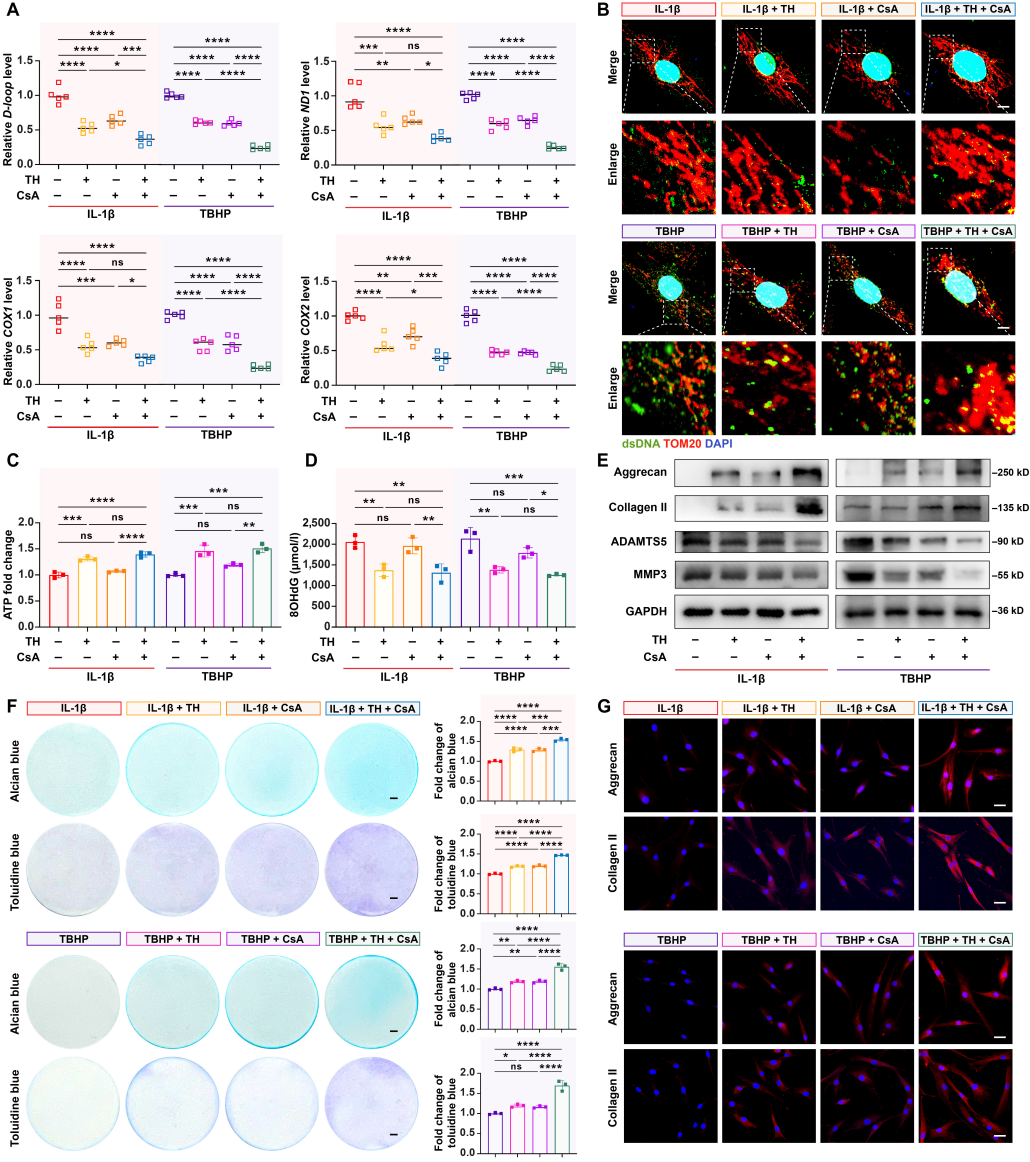
Combined OGG1 activation and mPTP blockade synergistically inhibited cytosolic mtDNA release and exerted chondroprotective effects in OA. (A) Relative cytosolic mtDNA amounts in HCs after TH or/and CsA treatment (*n* = 5). (B) Representative fluorescence images of dsDNA (green), TOM20 (red), and DAPI (blue) in above-treated HCs. Scale bars, 10 μm. (C) Relative quantification of ATP production in the above-treated HCs (*n* = 3). (D) 8OHdG concentrations measured by enzyme-linked immunosorbent assay in above-treated HCs (*n* = 3). (E) Western blotting results of aggrecan, collagen II, ADAMTS5, and MMP3 expression in above-treated HCs (*n* = 3). (F) Alcian blue and toluidine blue staining in above-treated HCs (left) and their quantification data (right) (*n* = 3). Scale bars, 5 μm. (G) Representative fluorescence images of aggrecan and collagen II in above-treated HCs (*n* = 3). Scale bars, 25 μm. All bar graphs: means ± SD. ****P < 0.0001, ***P < 0.001, **P < 0.01, *P < 0.05, and *P* > 0.05 (not significant [ns]).

Mechanistically, OGG1 activation facilitates the repair of ox-mtDNA lesions, whereas mPTP inhibition attenuates mtDNA extrusion through mitochondrial permeability channels. We hypothesized that combined OGG1 activation and mPTP inhibition would synergistically prevent pathological mtDNA cytosolic release. To verify this effect, we extracted cytosolic mtDNA after treating stimulated HCs with TH and/or CsA for 24 h. The results showed that all cytosolic mtDNA-encoded genes in the cotreatment group were decreased at varying degrees in comparison with the monotherapy groups (Fig. 4A). This observation was further confirmed by the fluorescence colocalization analysis of dsDNA and mitochondria (Fig. 4B).

Further, we assessed the impacts of TH and CsA combination treatment on mitochondrial energy metabolism and ox-mtDNA clearance. Interestingly, the combination treatment maintained the TH-mediated enhancement of ATP production (Fig. 4C) and ox-mtDNA clearance (Fig. 4D), showing its superior efficacy to CsA monotherapy. These results indicate that mPTP excessive opening inhibition does not directly contribute to mitochondrial energy metabolism or ox-mtDNA clearance, suggesting the complementary mechanisms of these dual agents. Extending these observations to cartilage metabolism, we found that both TH and CsA independently suppressed catabolism-related proteins (ADAMTS5 and MMP3) while preserving anabolism-related proteins (aggrecan and collagen II), with the combination treatment group showing the most pronounced effects (Fig. 4E and G and Fig. S8A and B). In addition, alcian blue and toluidine blue staining results confirmed that the combination treatment group attenuated ECM degradation more significantly (Fig. 4F).

Collectively, these findings imply that TH and CsA have functionally complementary effects. Simultaneous activation of OGG1 and inhibition of excessive mPTP opening suppress mtDNA cytosolic leakage more significantly and exert protective effects on OA cartilage.

### Combination of OGG1 activation and mPTP opening blockage inhibited cGAS-STING-NF-κB-mediated chondrocyte senescence

**Fig. 5.**
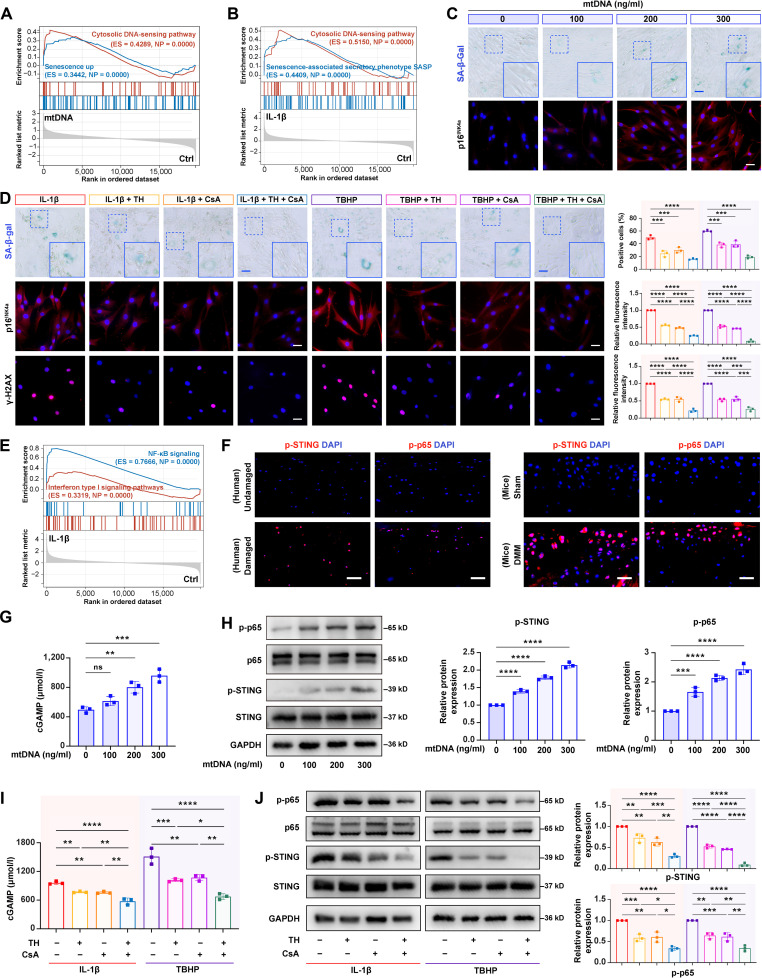
Combination of OGG1 activation and mPTP blocking inhibited cyclic guanosine monophosphate–adenosine monophosphate (cGAMP) synthase-stimulator of interferon genes-nuclear factor κB (cGAS-STING-NF-κB)-mediated chondrocyte senescence. (A) GSEA of mtDNA versus control groups (*n* = 3) with enrichment in “cytosolic DNA sensing” and “senescence up”. (B) GSEA of IL-1β versus control groups (*n* = 3) with enrichment in “cytosolic DNA sensing pathway” and “senescence associated secretory phenotype (SASP)”. (C) Senescence-associated β-galactosidase (SA-β-gal) staining and representative images of p16^INK4a^ showing mtDNA-induced senescence (*n* = 3). Scale bars, 100 μm (SA-β-gal staining) and 25 μm (p16^INK4a^ immunofluorescence [IF] images). (D) SA-β-gal staining and p16^INK4a^/γ-H2AX IF images showing therapeutic effects of TH or/and CsA (*n* = 3). Scale bars, 100 μm (SA-β-gal staining) and 25 μm (p16^INK4a^/γ-H2AX IF images). (E) GSEA of IL-1β versus control groups (*n* = 3) with enrichment in “NF-κB signaling” and “interferon type I signaling pathways”. (F) Representative images of phosphorylated STING (p-STING) and phosphorylated p65 (p-p65) in human and mouse OA cartilage (*n* = 3). Scale bars, 25 μm. (G) cGAMP concentrations in mtDNA-treated HCs (*n* = 3). (H) Western blotting analysis for p-p65, p65, p-STING, and STING expression levels in mtDNA-treated HCs and its quantification (*n* = 3). (I) cGAMP concentrations and (J) Western blotting analysis for p-p65, p65, p-STING, and STING expression levels with their quantification in stimulated HCs after TH or/and CsA treatment (*n* = 3). All bar graphs: means ± SD. ****P < 0.0001, ***P < 0.001, **P < 0.01, *P < 0.05, and *P* > 0.05 (not significant [ns]).

Although we demonstrated the impact of cytosolic mtDNA release on chondrocytes degradation, its underlying mechanisms remain incompletely understood. Previous studies indicated that mtDNA could function as a key regulator of SASP to promote cellular senescence [27,29,30]. Our RNA-seq analysis results demonstrated that senescence-related genes were significantly enriched in both mtDNA- and IL-1β-stimulated chondrocytes (Fig. 5A and B), suggesting that cytosolic mtDNA accumulation is associated with cellular senescence (Fig. S9). To validate this association, we examined senescence-related markers (SA-β-gal, p16^INK4a^, and γ-H2AX) and found that both mtDNA- and IL-1β-stimulated chondrocytes significantly increased the above indicators expression in chondrocytes (Fig. 5C and Figs. S10 and S11). Notably, although TH or CsA monotherapy only partially mitigated these senescence markers, the combination treatment produced significantly greater protection (Fig. 5D).

Previous studies showed that cytosolic mtDNA could promote senescence through cGAS–STING signaling, and the STING activation drove NF-κB activation toward chondrocyte degeneration [37,38]. Therefore, we hypothesized that cytosolic mtDNA release in chondrocytes might promote OA development by activating NF-κB through the cGAS-STING pathway. RNA-seq analysis results confirmed that there was a significant enrichment of the NF-κB signaling pathway in chondrocytes under stimulation (Fig. 5E and Fig. S12). Furthermore, our findings demonstrated that the expression levels of phosphorylated STING (p-STING) and phosphorylated p65 (p-p65) were markedly elevated in both degenerated human articular cartilage and DMM-induced mouse OA cartilage (Fig. 5F), providing direct evidence for cGAS-STING-NF-κB pathway activation. In addition, IL-1β, TBHP, or exogenous mtDNA stimulation equally increased the intracellular cGAMP concentrations in chondrocytes (Fig. 5G and Fig. S13A), providing evidence for cGAS enzymatic activation. Moreover, all 3 stimulations consistently up-regulated p-STING and p-p65 expression levels in chondrocytes (Fig. 5H and Figs. S13B and S14). Importantly, following mtDNA stimulation, treatment with the STING inhibitor C-176 (20 μM) markedly suppressed the activation of this pathway in HCs, inhibited the expression of senescence markers (Fig. S15A), and reversed the imbalance between anabolic and catabolic markers in chondrocytes (Fig. S15B). Subsequently, pharmacological intervention results showed that both TH and CsA monotherapies significantly reduced cGAMP levels, whereas the combination therapy exhibited a superior synergistic inhibition effect (Fig. 5I). Western blot and cellular fluorescence detection results further confirmed that the combined treatment suppressed p-STING and p-p65 expression more effectively than either stimulated controls or monotherapy groups (Fig. 5J and Fig. S16A and B).

Collectively, these findings reveal that the combination of OGG1 activation and mPTP inhibition effectively disrupts the cascaded activation of mtDNA-cGAS-STING-NF-κB signaling pathway, thereby significantly attenuating chondrocyte senescence progression.

### Activation of OGG1 and inhibition of mPTP attenuated OA progression in mice

**Fig. 6.**
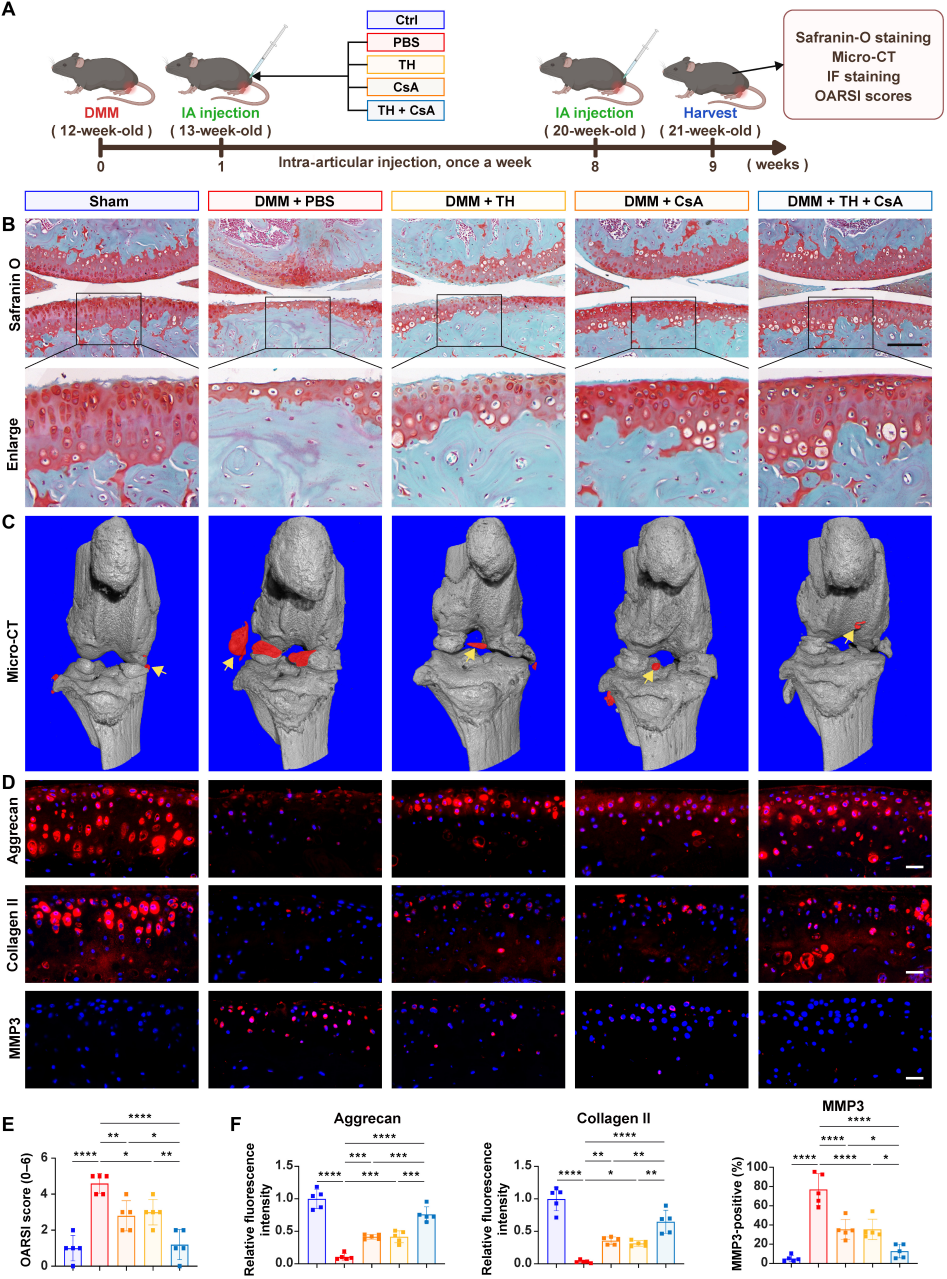
(A) Schematic diagram for the intra-articular (IA) injection of TH or/and CsA in OA mice weekly for 8 weeks, followed by joint analysis. (B) Safranin-O/fast green staining for the knee cartilage after different treatments for 8 weeks. Scale bar, 100 μm. (C) Micro-computed-tomography (micro-CT) assessment combined with 3-dimensional joint reconstruction in mice after different treatments. The region of interest (ROI) corresponding to periarticular osteophytes is delineated in red. (D) Representative immunofluorescence images (including aggrecan, collagen II, and MMP3) for different groups (*n* = 5). Scale bars, 25 μm. (E) Statistical analysis of Osteoarthritis Research Society International (OARSI) scores in (B) (*n* = 5). (F) Representative IF images of aggrecan, collagen II, and MMP3 (*n* = 5). All bar graphs: means ± SD. ****P < 0.0001, ***P < 0.001, **P < 0.01, and *P < 0.05.

For the purpose of assessing the therapeutic impacts of OGG1 activation and mPTP opening blockage in the experimental mice, 12-week-old mice were used to establish a right knee OA model and then categorized into 5 groups: 4 groups underwent DMM surgery and were subsequently given weekly intra-articular injections of phosphate-buffered saline (PBS), TH (10 mg/kg), CsA (10 mg/kg), or TH (10 mg/kg) + CsA (10 mg/kg) starting 1 week postsurgery for 8 consecutive weeks, with a sham group receiving neither DMM surgery nor injections (Fig. 6A).

After 8 weeks of treatment, hematoxylin and eosin staining of major organs showed no obvious impairment of liver or kidney function (Fig. S17). Safranin-O/fast green staining and micro-computed-tomography (micro-CT) analysis results revealed that both TH and CsA monotherapy groups showed partial reductions in articular cartilage damage and osteophyte formation in contrast to the PBS group, and the combination therapy group demonstrated superior chondroprotective and antiosteophytic effects, as further confirmed by Osteoarthritis Research Society International (OARSI) scoring (Fig. 6B, C, and E). Moreover, as opposed to the control group, monotherapy groups exhibited increased expression of anabolic markers (aggrecan and collagen II) and decreased catabolic marker (MMP3) expression, and the combination therapy showed more pronounced restoration of metabolic balance in OA cartilage (Fig. 6D and F).

**Fig. 7.**
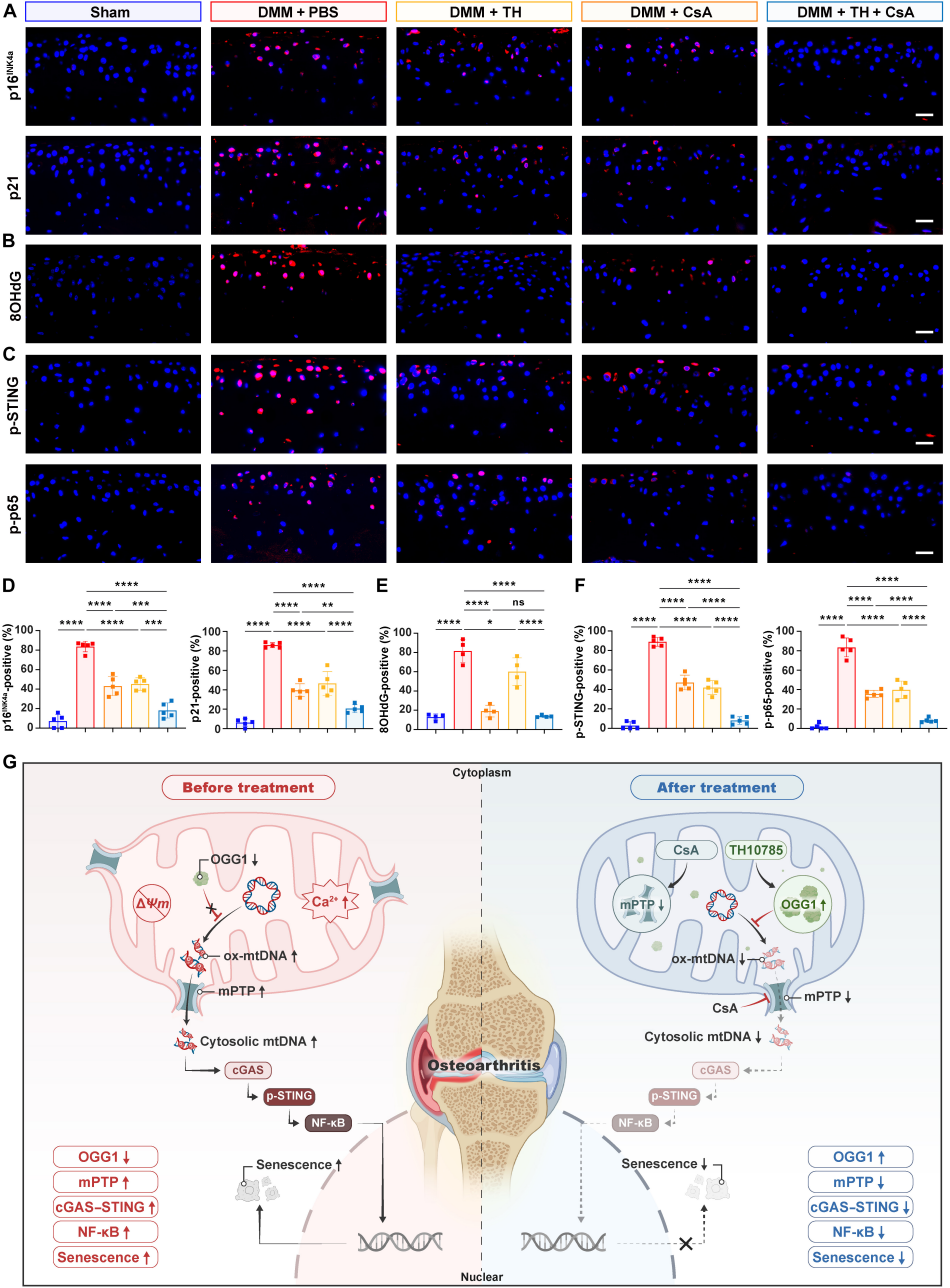
Activation of OGG1 and blockade of mPTP suppressed cGAS-STING-NF-κB-mediated cartilage senescence in OA. (A to F) Representative immunofluorescence images [including p16^INK4a^, p21 (A), 8OHdG (B), phosphorylated STING (p-STING), and phosphorylated p65 (p-p65) (C)] for different groups and representative immunofluorescence analysis [including p16^INK4a^, p21 (D), 8OHdG (E), p-STING, and p-p65 (F)] (*n* = 5). Scale bars, 25 μm. (G) Schematic diagram of the combined targeting strategy for OGG1 and mPTP: new insights into developing mtDNA-regulated targeted therapeutics for OA. All bar graphs: means ± SD. ****P < 0.0001, ***P < 0.001, **P < 0.01, *P < 0.05, and *P* > 0.05 (not significant [ns]).

Mechanistically, both TH and CsA suppressed senescence markers (p16^INK4a^ and p21) expression in OA cartilage, with enhanced effects in the combination treatment group (Fig. 7A and D). Consistent with the in vitro findings, although CsA showed a limited effect on 8OHdG levels (Fig. 7B and E), all drug treatments suppressed p-STING and p-p65 expression in mice cartilage, and the combination therapy showed the most profound inhibition (Fig. 7C and F).

These results collectively demonstrate that the combination of mtDNA repair promotion via OGG1 activation and mtDNA release inhibition via mPTP blockade synergistically suppresses the cGAS-STING-NF-κB axis, thereby mitigating chondrocyte senescence and preventing OA progression (Fig. 7G).

## Discussion

Our research elucidated the interplay between cytosolic mtDNA accumulation and cartilage metabolism/cellular senescence, a critical yet underexplored axis in OA pathogenesis. We confirmed that inflammatory or oxidative stress stimuli increase cytosolic mtDNA levels, driving cartilage ECM degradation, which was further validated by exogenous mtDNA intervention experiments. Cytosolic mtDNA is recognized as DAMPs promoting proinflammatory cytokine release, yet its association with cellular senescence remains uncharacterized [30,39]. Our study showed that it up-regulated SASP expression and senescence-associated DNA damage in chondrocytes, highlighting its pathogenic role in OA.

The stability of mitochondrial nucleic acids and the homeostasis of enzymes are both indispensable for sustaining mitochondrial function. For instance, lon protease homolog (LONP)-mediated regulation of mitochondrial proteostasis can delay bone loss [40], while mtDNA can drive disease progression when its structure or function is abnormal. Previous studies have confirmed that the cleavage and fragmentation of mtDNA are involved in regulating its efflux into the cytoplasm, which highlights the critical significance of mtDNA integrity [6–8]. OGG1, which recognizes and excises 8OHdG, maintains mtDNA integrity in OA. In our study, the OGG1 activator TH preserved mtDNA integrity by reducing ox-mtDNA generation, promoting ATP production, and inhibiting mtROS generation. In addition, OGG1 activation effectively reduced the content of cytosolic ox-mtDNA, suggesting that mtDNA release is associated with the regulatory mechanism of its damage repair integrity.

Notably, mitochondria, as highly dynamic organelles, have functional states that are crucial for mtDNA translocation. Mito-Ca^2+^ overload and Δ*Ψm* imbalance trigger excessive mPTP opening, which, in turn, promotes mtDNA efflux into the cytoplasm [18,41]. However, in this study, the treatment of TH failed to reverse these mitochondrial dysfunctions, but cotreatment with the mPTP inhibitor CsA significantly reduced cytosolic mtDNA, alleviating chondrocyte senescence and cartilage degradation. These results indicate that mtDNA release depends on both integrity regulation and mPTP-mediated translocation.

Importantly, both IL-1β and TBHP stimulations down-regulated OGG1 expression in a dose- and time-dependent manner, with a more pronounced effect observed under TBHP stimulation. Accordingly, cotreatment more effectively reduced 8OHdG levels in TBHP-stimulated HCs, suggesting that oxidative stress more directly impairs mtDNA repair and that mtDNA oxidative damage is a key driver of cartilage degeneration. Although no significant difference was observed in mPTP opening between IL-1β and TBHP groups, cotreatment more strongly inhibited mPTP opening and preserved Δ*Ψm* under IL-1β stimulation. These results indicate that mPTP-mediated mitochondrial dysfunction acts as an important contributor in the inflammatory microenvironment.

In addition, hyperactivation of the cGAS-STING-NF-κB axis was detected in both the DMM-induced mice and IL-1β-, TBHP-, mtDNA-stimulated chondrocytes, highlighting the essential function of the innate immune system in mtDNA-release-associated OA pathogenesis. Notably, exogenous non-ox-mtDNA still activated this axis, confirming OGG1’s primary role in maintaining mtDNA integrity rather than merely inhibiting oxidation. Growing evidence indicates that OA, similar to rheumatoid arthritis, is classified as a chronic inflammatory autoimmune disease and targeted intervention of its associated immune system can significantly improve therapeutic efficacy [42]. In our study, moreover, cotreatment with CsA further suppressed axis activation in both cellular and animal models, indicating that inhibition of the immune system in OA can further delay disease progression.

In conclusion, this research explored the effect of inflammatory or oxidative-stress-induced cytosolic mtDNA accumulation on cartilage metabolism and cellular senescence. We confirmed that OGG1 activation and mPTP blockade alleviate cartilage degradation and senescence by reducing cytosolic mtDNA, with distinct roles in synergistically suppressing mtDNA-induced cGAS-STING-NF-κB axis activation. Limitations include the inability to directly detect mtDNA efflux in DMM-induced mice, unaddressed potential effects of the OGG1 activator TH on nuclear OGG1, and focusing solely on the mPTP inhibitor CsA without validation of other mitochondrial pore inhibitors, which will be systematically explored further. Collectively, this study demonstrates the key function of cytosolic mtDNA in the pathological development of OA, demonstrating that mtDNA integrity and translocation collectively regulate cytosolic mtDNA content in OA, while combined targeting of OGG1 and mPTP provides new insights for developing mtDNA-regulated targeted OA therapeutics.

## Materials and Methods

### Sources of reagents and materials

TH, CsA, and C-176 were obtained from MedChemExpress (Shanghai, China). MitoTracker Green, JC-1 staining, ATP detecting kit, mitochondria/cytosol fractionation kit, and mPTP assay kit were obtained from Beyotime (Jiangsu, China). MitoSOX Red and Rhod2/AM were obtained from Invitrogen (Shanghai, China). IL-1β was obtained from Sino Biological Co. Ltd. (Beijing, China). TBHP was obtained from Merck (Darmstadt, Germany). DNeasy blood and tissue kit was obtained from QIAGEN (Beijing, China). Cell culture reagents, including fetal bovine serum, Dulbecco,s modified Eagle medium/nutrient mixture F-12, antibiotics (penicillin–streptomycin), and trypsin–ethylenediaminetetraacetic acid were purchased from Sigma-Aldrich (St. Louis, MO, USA). Enzyme-linked immunosorbent assay kits were obtained from Guangzhou Meilun Biotechnology Co. Ltd. (Guangzhou, China).

Antibodies including anti-aggrecan (catalog no. ab3778), anti-collagen II (catalog no. ab307674), and anti-dsDNA (catalog no. ab27156) were from Abcam (Cambridge, UK); anti-MMP3 (catalog no. 17873-1-AP), anti-OGG1 (catalog no. 30302-1-AP), anti-p-p65 (catalog no. 82335-1-RR), anti-p65 (catalog no. 80979-1-RR), anti-glyceraldehyde-3-phosphate dehydrogenase (GAPDH; catalog no. 60004-1-Ig), anti-aggrecan (catalog no. 13880-1-AP), anti-collagen II (catalog no. 28459-1-AP), anti-p16^INK4a^ (catalog no. 10883-1-AP), anti-p21 (catalog no. 10355-1-AP), and anti-TOM20 (catalog no. 11802-1-AP) were from Proteintech (Wuhan, China); anti-p-STING (catalog no. TA7416S) and anti-STING (catalog no. T57204) were from Abmart (Shanghai, China); anti-8OHdG (catalog no. bs-1278R) was from Bioss (Beijing, China).

### Human cartilage collection, chondrocyte isolation, and assays

The HCs utilized in this study were obtained from the knee cartilage of patients with OA, with ethical approval from the Ethics Committee of Zhujiang Hospital, Southern Medical University (no. 2019-KY-022-03). These HCs were isolated via trypsin and type II collagenase digestion, maintained in Dulbecco’s modified Eagle’s medium/F12 medium with 10% fetal bovine serum, and third-generation cells were inoculated into 6-well plates with IL-1β (10 ng/ml) or TBHP (80 μM) with or without TH (1 μM) or/and CsA (1 μM) for 24-h stimulation.

To comparatively quantify cytoplasmic mtDNA, we used the mitochondria/cytosol fractionation kit for the isolation of cytosolic components [43]. The DNeasy blood and tissue kit was used for DNA extraction from both whole-cell preparations and cytosolic fractions. RT-qPCR was carried out on the DNA samples from whole-cell/cytosolic fractions with mtDNA primers, which were listed in the Table S1. mtDNA quantification cycle values derived from total cellular extracts were utilized as internal reference standards for normalizing the mtDNA levels detected in cytosolic extracts.

The construction of RNA-seq libraries and high-throughput RNA-seq were carried out by Shanghai Bohao Biotechnology Co. Ltd. (China) with the BGISEQ-500 high-throughput sequencer. In addition, each group included 3 biological replicates (*n* = 3).

### Synthesis of exogenous mtDNA

The mtDNA was synthesized by Guangzhou IGE Biotechnology Co. Ltd. A 150-bp fragment within the D-loop region of mtDNA was selected for subsequent experiments. The specific sequence of this 150-bp D-loop fragment is G​ATC​ACA​GGT​CTA​TCA​CCC​CTA​TTA​ACC​ACT​CAC​GGG​AGC​TCT​CCA​TGC​ATT​TGG​TAT​TTT​CGT​CTG​GGG​GGG​TAT​GCA​CGC​GAT​AGC​ATT​TGC​GAG​ACG​CTG​GAG​CCG​GAG​CAC​CCT​ATGTCGCAGTATCTGTCTTTGATTCCTGCCTCATC.

### Cell Counting Kit-8 viability assay

For the purpose of evaluating how varying drug concentrations influence cell viability, the Cell Counting Kit-8 assay was performed in this study, with the half-maximal inhibitory concentration subsequently calculated. Briefly, ~5,000 HCs were seeded into 96-well plates and cultured for 24 or 48 h. Following this, the cells were exposed to serial concentrations (0, 200, 400, 600, 800, and 1000 nM) of TH or CsA for identical 24- or 48-h incubation periods. After removing the original culture medium, fresh medium supplemented with 10% Cell Counting Kit-8 solution (Dojindo, Japan) was dispensed into each well, and the plates were placed in a 37 °C incubator. A microplate reader (BioTek, USA) was used to measure the absorbance at 450 nm. Based on the resulting viability curves, optimal drug concentrations were selected for subsequent experimental procedures.

### RNA extraction and RT-qPCR

Total RNA from HCs was isolated using TRIzol kit (Thermo Fisher Scientific, NY, USA) per the manufacturer’s instructions. For mRNA expression profiling, 1000 ng of the isolated total RNA was reverse-transcribed with PrimeScript RT Master Mix (Thermo Fisher Scientific, NY, USA) to detect mRNA expression. RT-qPCR was run on the LightCycler480 system (Roche, Basel, Switzerland) using SYBR Premix Ex Taq II (Thermo Fisher Scientific, NY, USA). The expression levels of target genes (listed in the Supplementary Materials) were normalized against the housekeeping gene GAPDH, and the relative mRNA abundance was calculated utilizing the 2^−ΔΔCq^ method.

### Western blotting

Total cellular proteins were isolated from HCs with radioimmunoprecipitation assay lysis buffer (Beyotime, China). Bicinchoninic acid assay was used for the quantification of protein concentrations. Equal quantities of protein lysates were separated via 10% or 12% sodium dodecyl sulfate–polyacrylamide gel electrophoresis, followed by electrotransfer onto polyvinylidene difluoride membranes (Merck Millipore, Germany). Blocking of the membranes was carried out using 5% nonfat dry milk in tris-buffered saline at room temperature for a period of 1 h, prior to overnight incubation with primary antibodies. The membranes were treated with horseradish-peroxidase-conjugated secondary antibodies for 1 h following washes with tris-buffered saline containing 0.1% Tween 20. Immunoreactive bands were visualized using enhanced chemiluminescence detection reagents (Merck Millipore, Germany) and imaged on a chemiluminescence image system (Bio-Rad, USA), with densitometry analyzed via Image Lab software.

### mtROS level, mito-Ca^2+^ level, and Δ*Ψm* detection

The mtROS levels were assessed using MitoSOX dye. Briefly, HCs were pretreated with TH or CsA for 6 h and then exposed to IL-1β or TBHP. Following the treatment, HCs were stained with MitoSOX for 40 min and Hoechst 33258 for 10 min, and mtROS levels were analyzed using a fluorescence microscope (Nikon Ti2-E, Tokyo, Japan).

For determining mito-Ca^2+^ content, the cell-permeable fluorescent Ca^2+^ indicator Rhod-2/AM was used for relative quantitative analysis of intracellular calcium levels after different treatments. Samples were coincubated with MitoTracker and Rhod-2/AM (5 μM) at 37 °C over a period of 25 min and then examined with a fluorescence microscope after washing.

To evaluate mitochondrial functional phenotypes in HCs after various treatments, the culture medium was thoroughly discarded. Medium containing JC-1 solution was added after washing with PBS. Following a 30-min incubation at 37 °C, we discarded the supernatant and rinsed the cells with a buffer specifically formulated for JC-1 staining. These samples were then examined using a fluorescence microscopy system for image acquisition.

### TEM detection

To detect mitochondrial morphology in chondrocytes postinflammatory stimulation, HCs were fixed overnight at 4 °C in a TEM fixative solution. After this primary fixation step, the cells were processed through a series of sequential procedures: postfixation with 1% osmium tetroxide, staining with uranyl acetate, gradient dehydration in ethanol solutions, and final embedding in epoxy resin. After mounting and counterstaining the ultrathin sections with uranyl acetate and lead citrate, all morphological observations were performed using a Hitachi HT-7800 TEM to acquire high-resolution images.

### mPTP assay

mPTP opening activity was evaluated after a 24-h cell culture period in the designated medium. Briefly, HCs were pretreated with TH or CsA for 6 h and then exposed to IL-1β or TBHP. Following the abovementioned treatment, the mPTP assay was performed in strict adherence to the kit manufacturer’s protocols, and the processed samples were finally visualized under a fluorescence microscope.

### Alcian blue, toluidine blue staining, and SA-β-gal staining

After different treatments, HCs were plated in 12-well plates. After fixation with 4% paraformaldehyde and PBS rinses, cells were subjected to staining with alcian blue and toluidine blue (Solarbio, China) for 15 min, respectively. After removing residual stain, images were acquired via a light microscope.

Senescence-positive HCs were identified using a SA-β-gal detecting kit (Beyotime, China). Following a 15-min room-temperature fixation step with β-galactosidase fixative, cells were rinsed with PBS and subjected to overnight incubation at 37 °C with SA-β-gal staining reagent. The number of senescent-positive HCs was determined via microscopic observation and counting.

### Animal experiments

The animal studies conducted in this research were authorized by the Laboratory Animal Ethics Committee of Zhujiang Hospital, Southern Medical University (LAEC-2023-148). Twelve-week-old male C57BL/6 mice were arbitrarily sorted into 5 experimental groups: (a) sham, (b) DMM + PBS, (c) DMM + TH (10 mg/kg), (d) DMM + CsA (10 mg/kg), and (e) DMM + TH (10 mg/kg) + CsA (10 mg/kg). Each mouse received a 10-μl intra-articular injection. To establish OA model, mice were anesthetized with 3% isoflurane in 97% oxygen using a controlled evaporator (VMR, Matrx, USA) prior to DMM surgery. During surgery, the right knee joint was visualized under a stereomicroscope via a medial capsular incision. Medial displacement of the medial meniscus was performed, followed by resection of the medial meniscotibial ligament and primary suture closure of the incision. All mice were euthanized at 12 weeks postsurgery or first injection, and right knees were harvested for further evaluation.

### Micro-CT analysis

Micro-CT analysis was carried out on fixed knee joint specimens at a resolution of 96 μm, and the assay was performed by Guangzhou Zhongke Kaison Medical Technology Co. Ltd. Three-dimensional reconstruction and image acquisition of the knee joint were performed using the software 3D-MED 3.0. Periarticular osteophytes were designated as the region of interest (ROI) and manually segmented using Mimics 5.0 software. To minimize selection bias, manual segmentation was independently performed by 2 blinded observers, and the osteophyte ROIs were labeled in red.

### Histological and immunofluorescence analyses

Following fixation in 4% paraformaldehyde, all specimens were subjected to decalcification in EDTA solution. Paraffin-embedded samples for Safranin-O/fast green staining were sectioned at 4 μm. According to the OARSI guideline, cartilage specimens derived from femoral condyles and tibial plateaus were scored separately (0 = normal to 6 ≥ 75% articular surface erosion).

For immunofluorescence, the sections were dewaxed conventionally and then repaired in EDTA solution at 65 °C for 12 h. The slices were first treated with 0.5% Triton X-100 for membrane permeabilization, followed by blocking with 5% bovine serum albumin prior to overnight incubation with primary antibodies. The sections were treated with Alexa Fluor 555-conjugated fluorescent secondary antibodies, and cell nuclei were counterstained with 4′,6-diamidino-2-phenylindole (DAPI) after PBS washes. Finally, we captured fluorescent images using a fluorescence microscope system.

For quantification, the proteins primarily localized to the cartilage matrix (such as aggrecan and collagen II) were analyzed by selecting and comparing the cartilage matrix regions across samples. As for proteins distributed in the cytoplasm or throughout the cell, the whole cell was designated as the ROI for analysis.

### Statistical analysis

All experimental data were expressed as means ± standard deviation (SD) or means ± standard error of the mean (SEM), with results derived from no fewer than 3 independent experimental replicates. For comparisons between 2 distinct groups, the unpaired Student’s *t* test was adopted, whereas multigroup comparisons were implemented via one-way analysis of variance (ANOVA), followed by post hoc pairwise comparisons with the least significant difference test or the Mann–Whitney *U* test as appropriate. All statistical computations were executed utilizing the SPSS 22.0 software package (IBM Corporation, Chicago, USA). ****P < 0.0001, ***P < 0.001, **P < 0.01, *P < 0.05, and *P* > 0.05 (not significant [ns]).

## Ethical Approval

The HCs utilized in this study were obtained from the knee cartilage of patients with OA, with ethical approval from the Ethics Committee of Zhujiang Hospital, Southern Medical University (no. 2019-KY-022-03). All patients provided written informed consent before the operative procedure. The animal studies conducted in this research were authorized by the Laboratory Animal Ethics Committee of Zhujiang Hospital, Southern Medical University (LAEC-2023-148).

## Data Availability

The experimental data can be obtained from the corresponding author upon reasonable request.

## References

[1] Tang S, Zhang C, Oo WM, Fu K, Risberg MA, Bierma-Zeinstra SM, Neogi T, Atukorala I, Malfait A-M, Ding C, et al. Osteoarthritis. Nat Rev Dis Primers. 2025;11(1):10. 39948092 10.1038/s41572-025-00594-6

[2] Chen L, Yang J, Cai Z, Huang Y, Xiao P, Chen H, Luo X, Huang W, Cui W, Hu N. Mitochondrial-oriented injectable hydrogel microspheres maintain homeostasis of chondrocyte metabolism to promote subcellular therapy in osteoarthritis. Research. 2024;7:0306. 38274127 10.34133/research.0306 PMC10809599

[3] Xu X, Pang Y, Fan X. Mitochondria in oxidative stress, inflammation and aging: From mechanisms to therapeutic advances. Signal Transduct Target Ther. 2025;10(1):190. 40500258 10.1038/s41392-025-02253-4 PMC12159213

[4] Sun L, Wang Y, Kan T, Wang H, Cui J, Wang L, Liu C, Li H, Yu Z, Yan M. Elevated expression of Piezo1 activates the cGAS-STING pathway in chondrocytes by releasing mitochondrial DNA. Osteoarthritis Cartilage. 2025;33(5):601–615. 39978573 10.1016/j.joca.2025.02.778

[5] Zhang W, Li G, Luo R, Lei J, Song Y, Wang B, Ma L, Liao Z, Ke W, Liu H, et al. Cytosolic escape of mitochondrial DNA triggers cGAS-STING-NLRP3 axis-dependent nucleus pulposus cell pyroptosis. Exp Mol Med. 2022;54(2):129–142. 35145201 10.1038/s12276-022-00729-9 PMC8894389

[6] Xian H, Watari K, Sanchez-Lopez E, Offenberger J, Onyuru J, Sampath H, Ying W, Hoffman HM, Shadel GS, Karin M. Oxidized DNA fragments exit mitochondria via mPTP- and VDAC-dependent channels to activate NLRP3 inflammasome and interferon signaling. Immunity. 2022;55(8):1370–1385.e8. 35835107 10.1016/j.immuni.2022.06.007 PMC9378606

[7] Sharma P, Sampath H. Mitochondrial DNA integrity: Role in health and disease. Cells. 2019;8(2):100. 30700008 10.3390/cells8020100 PMC6406942

[8] Muftuoglu M, Mori MP, de Souza-Pinto NC. Formation and repair of oxidative damage in the mitochondrial DNA. Mitochondrion. 2014;17:164–181. 24704805 10.1016/j.mito.2014.03.007

[9] Fontana GA, Gahlon HL. Mechanisms of replication and repair in mitochondrial DNA deletion formation. Nucleic Acids Res. 2020;48(20):11244–11258. 33021629 10.1093/nar/gkaa804 PMC7672454

[10] He Y, Ding Q, Chen W, Lin C, Ge L, Ying C, Xu K, Wu Z, Xu L, Ran J, et al. LONP1 downregulation with ageing contributes to osteoarthritis via mitochondrial dysfunction. Free Radic Biol Med. 2022;191:176–190. 36064070 10.1016/j.freeradbiomed.2022.08.038

[11] Zhong Y, Zhang X, Feng R, Fan Z, Zhang Z, Zhang Q-W, Wan J-B, Wang Y, Yu H, Li G. OGG1: An emerging multifunctional therapeutic target for the treatment of diseases caused by oxidative DNA damage. Med Res Rev. 2024;44(6):2825–2848. 39119702 10.1002/med.22068

[12] Kim J, Xu M, Xo R, Mates A, Wilson GL, Pearsall AW IV, Grishko V. Mitochondrial DNA damage is involved in apoptosis caused by pro-inflammatory cytokines in human OA chondrocytes. Osteoarthritis Cartilage. 2010;18(3):424–432. 19822235 10.1016/j.joca.2009.09.008

[13] Tumurkhuu G, Shimada K, Dagvadorj J, Crother TR, Zhang W, Luthringer D, Gottlieb RA, Chen S, Arditi M. Ogg1-dependent DNA repair regulates NLRP3 inflammasome and prevents atherosclerosis. Circ Res. 2016;119(6):e76–e90. 27384322 10.1161/CIRCRESAHA.116.308362 PMC5010464

[14] Hussain M, Chu X, Sahbaz BD, Gray S, Pekhale K, Park J-H, Croteau DL, Bohr VA. Mitochondrial OGG1 expression reduces age-associated neuroinflammation by regulating cytosolic mitochondrial DNA. Free Radic Biol Med. 2023;203:34–44. 37011700 10.1016/j.freeradbiomed.2023.03.262 PMC10247526

[15] Kim J, Gupta R, Blanco LP, Yang S, Shteinfer-Kuzmine A, Wang K, Zhu J, Yoon HE, Wang X, Kerkhofs M, et al. VDAC oligomers form mitochondrial pores to release mtDNA fragments and promote lupus-like disease. Science. 2019;366(6472):1531–1536. 31857488 10.1126/science.aav4011 PMC8325171

[16] Chen L, Dong J, Liao S, Wang S, Wu Z, Zuo M, Liu B, Yan C, Chen Y, He H, et al. Loss of Sam50 in hepatocytes induces cardiolipin-dependent mitochondrial membrane remodeling to trigger mtDNA release and liver injury. Hepatology. 2022;76(5):1389–1408. 35313046 10.1002/hep.32471

[17] Yu C-H, Davidson S, Harapas CR, Hilton JB, Mlodzianoski MJ, Laohamonthonkul P, Louis C, Low RRJ, Moecking J, De Nardo D, et al. TDP-43 triggers mitochondrial DNA release via mPTP to activate cGAS/STING in ALS. Cell. 2020;183(3):636–649.e18. 33031745 10.1016/j.cell.2020.09.020 PMC7599077

[18] NavaneethaKrishnan S, Rosales JL, Lee K-Y. mPTP opening caused by Cdk5 loss is due to increased mitochondrial Ca^2+^ uptake. Oncogene. 2020;39(13):2797–2806. 32024968 10.1038/s41388-020-1188-5 PMC7098883

[19] Huang LS, Hong Z, Wu W, Xiong S, Zhong M, Gao X, Rehman J, Malik AB. mtDNA activates cGAS signaling and suppresses the YAP-mediated endothelial cell proliferation program to promote inflammatory injury. Immunity. 2020;52(3):475–486.e5. 32164878 10.1016/j.immuni.2020.02.002 PMC7266657

[20] Yan Z, He Z, Jiang H, Zhang Y, Xu Y, Zhang Y. TRPV4-mediated mitochondrial dysfunction induces pyroptosis and cartilage degradation in osteoarthritis via the Drp1-HK2 axis. Int Immunopharmacol. 2023;123: Article 110651. 37506502 10.1016/j.intimp.2023.110651

[21] Endlicher R, Drahota Z, Štefková K, Červinková Z, Kučera O. The mitochondrial permeability transition pore-current knowledge of its structure, function, and regulation, and optimized methods for evaluating its functional state. Cells. 2023;12(9):1273. 37174672 10.3390/cells12091273 PMC10177258

[22] Hernandez-Segura A, de Jong TV, Melov S, Guryev V, Campisi J, Demaria M. Unmasking transcriptional heterogeneity in senescent cells. Curr Biol. 2017;27(17):2652–2660.e4. 28844647 10.1016/j.cub.2017.07.033 PMC5788810

[23] Rossiello F, Jurk D, Passos JF, d’Adda di Fagagna F. Telomere dysfunction in ageing and age-related diseases. Nat Cell Biol. 2022;24(2):135–147. 35165420 10.1038/s41556-022-00842-x PMC8985209

[24] Finkel T, Holbrook NJ. Oxidants, oxidative stress and the biology of ageing. Nature. 2000;408(6809):239–247. 11089981 10.1038/35041687

[25] Coryell PR, Diekman BO, Loeser RF. Mechanisms and therapeutic implications of cellular senescence in osteoarthritis. Nat Rev Rheumatol. 2021;17(1):47–57. 33208917 10.1038/s41584-020-00533-7 PMC8035495

[26] Pinto M, Moraes CT. Mechanisms linking mtDNA damage and aging. Free Radic Biol Med. 2015;85:250–258. 25979659 10.1016/j.freeradbiomed.2015.05.005 PMC4508218

[27] Victorelli S, Salmonowicz H, Chapman J, Martini H, Vizioli MG, Riley JS, Cloix C, Hall-Younger E, Espindola-Netto JM, Jurk D, et al. Apoptotic stress causes mtDNA release during senescence and drives the SASP. Nature. 2023;622(7983):627–636. 37821702 10.1038/s41586-023-06621-4 PMC10584674

[28] Newman LE, Shadel GS. Mitochondrial DNA release in innate immune signaling. Annu Rev Biochem. 2023;92:299–332. 37001140 10.1146/annurev-biochem-032620-104401 PMC11058562

[29] Glück S, Guey B, Gulen MF, Wolter K, Kang T-W, Schmacke NA, Bridgeman A, Rehwinkel J, Zender L, Ablasser A. Innate immune sensing of cytosolic chromatin fragments through cGAS promotes senescence. Nat Cell Biol. 2017;19(9):1061–1070. 28759028 10.1038/ncb3586 PMC5826565

[30] de Galarreta MR, Lujambio A. DNA sensing in senescence. Nat Cell Biol. 2017;19(9):1008–1009. 28855731 10.1038/ncb3603

[31] Hu M-M, Shu H-B. Mitochondrial DNA-triggered innate immune response: Mechanisms and diseases. Cell Mol Immunol. 2023;20(12):1403–1412. 37932533 10.1038/s41423-023-01086-x PMC10687031

[32] Riley JS, Tait SW. Mitochondrial DNA in inflammation and immunity. EMBO Rep. 2020;21(4): Article e49799. 32202065 10.15252/embr.201949799 PMC7132203

[33] Zhu J-Y, Chen M, Mu W-J, Luo H-Y, Guo L. Higd1a facilitates exercise-mediated alleviation of fatty liver in diet-induced obese mice. Metabolism. 2022;134: Article 155241. 35750235 10.1016/j.metabol.2022.155241

[34] Michel M, Benítez-Buelga C, Calvo PA, Hanna BMF, Mortusewicz O, Masuyer G, Davies J, Wallner O, Sanjiv K, Albers JJ, et al. Small-molecule activation of OGG1 increases oxidative DNA damage repair by gaining a new function. Science. 2022;376(6600):1471–1476. 35737787 10.1126/science.abf8980

[35] Ouyang W, Wang S, Yan D, Wu J, Zhang Y, Li W, Hu J, Liu Z. The cGAS-STING pathway-dependent sensing of mitochondrial DNA mediates ocular surface inflammation. Signal Transduct Target Ther. 2023;8(1):371. 37735446 10.1038/s41392-023-01624-z PMC10514335

[36] Li X, Kong D, Yu Q, Si X, Yang L, Zeng X, Li Y, Shi J, Qian P, Huang H, et al. Cyclosporine a regulates PMN-MDSCs viability and function through MPTP in acute GVHD: Old medication, new target. Transplant Cell Ther. 2022;28(7):411.e1–411.e9. 10.1016/j.jtct.2022.04.010 35430420

[37] Sun K, Zhang X, Hou L, Lu F, Liu H, Zheng Z, Guo Z, Xu J, Ruan Z, Hou Y, et al. TRPM2-mediated feed-forward loop promotes chondrocyte damage in osteoarthritis via calcium-cGAS-STING-NF-κB pathway. J Adv Res. 2024;75:213–217. 39505144 10.1016/j.jare.2024.11.007 PMC12536589

[38] Kim J, Kim H-S, Chung JH. Molecular mechanisms of mitochondrial DNA release and activation of the cGAS-STING pathway. Exp Mol Med. 2023;55(3):510–519. 36964253 10.1038/s12276-023-00965-7 PMC10037406

[39] Long G, Gong R, Wang Q, Zhang D, Huang C. Role of released mitochondrial DNA in acute lung injury. Front Immunol. 2022;13: Article 973089. 36059472 10.3389/fimmu.2022.973089 PMC9433898

[40] Jin Z, Mao Y, Guo Q, Yin Y, Kiram A, Zhou D, Yang J, Zhou Z, Xue J, Feng Z, et al. Imbalanced skeletal muscle mitochondrial proteostasis causes bone loss. Research. 2024;7:0465. 39221030 10.34133/research.0465 PMC11362843

[41] Bernardi P, Gerle C, Halestrap AP, Jonas EA, Karch J, Mnatsakanyan N, Pavlov E, Sheu S-S, Soukas AA. Identity, structure, and function of the mitochondrial permeability transition pore: Controversies, consensus, recent advances, and future directions. Cell Death Differ. 2023;30(8):1869–1885. 37460667 10.1038/s41418-023-01187-0 PMC10406888

[42] Zhang C, Ma P, Qin A, Wang L, Dai K, Liu Y, Zhao J, Lu Z. Current immunotherapy strategies for rheumatoid arthritis: The immunoengineering and delivery systems. Research. 2023;6:0220. 39902178 10.34133/research.0220 PMC11789687

[43] Zhong W, Rao Z, Xu J, Sun Y, Hu H, Wang P, Xia Y, Pan X, Tang W, Chen Z, et al. Defective mitophagy in aged macrophages promotes mitochondrial DNA cytosolic leakage to activate STING signaling during liver sterile inflammation. Aging Cell. 2022;21(6): Article e13622. 35599014 10.1111/acel.13622 PMC9197407

