# Supplementary material for: Mitochondrial 8-Oxoguanine DNA Glycosylase 1–Mitochondrial Permeability Transition Pore Axis Drives Mitochondrial DNA Escape and Accelerates Osteoarthritis Progression
Source: Research (Wash D C). 2026 Apr 15;9:1235. doi: 10.34133/research.1235 (PMC13080097; doi:10.34133/research.1235)
Supplement: Supplementary 1 — Table S1 Figs. S1 to S17 [file research.1235.f1.docx]

**Supplementary Materials**

**Mitochondrial OGG1-mPTP Axis Drives mtDNA Escape and Accelerates Osteoarthritis Progression**

Shiqian Huang^1^†, Heting Yu^1^†, Weizhong Qi^1^†, Na Lin^1^, Jianmao Chen^1^, Hong Huang^1^, Pengcheng Hu^1^, Ziqi Zhou^1^, Mengdi Zhang^1^, Guangfeng Ruan^2*^, Song Xue^3*^, and Changhai Ding^1,4,5*^

^1^Clinical Research Centre, Zhujiang Hospital, Southern Medical University, Guangzhou, China.

^2^Clinical Research Center, Guangzhou First People’s Hospital, Guangzhou Medical University, Guangzhou, China.

^3^Department of Sports Medicine and Rehabilitation, Peking University Shenzhen Hospital, Shenzhen Peking University-The Hong Kong University of Science and Technology Medical Center, Shenzhen, China.

^4^Menzies Institute for Medical Research, University of Tasmania, Hobart, Australia.

^5^Clinical Research Centre, Beijing Tsinghua Changgung Hospital, Tsinghua Medicine, Tsinghua University, Beijing, China.

*Address correspondence to: Guangfeng Ruan; [ruan1989.ok@163.com](mailto:ruan1989.ok@163.com) and Song Xue; [freexuesong@163.com](mailto:freexuesong@163.com) and Changhai Ding; [changhai.ding@utas.edu.au](mailto:changhai.ding@utas.edu.au)

†These authors contributed equally to this work.

**1. Supplementary Table 1: gene primer sequences**

| Gene | Primer forward | Primer reverse |
| --- | --- | --- |
| *OGG1* | ATTCCAAGGTGTGCGACTGCTG | GATGCGGGCGATGTTGTTGTTG |
| *FEN1* | CCATCCGTGAGAATGACATCAAGAG | CGAACAGCAATCAGGAACTGGTAG |
| *NTHL1* | CAAGATGGCACACCTGGCTATGG | GTTGCCTTCTTGGTCCACCTCAG |
| *PRDX3* | AGTTGTCGCAGTCTCAGTGGATTC | CCTTCTAACAGCACACCGTAGTCTC |
| *PARP1* | CCAGGTCAAGGAGGAAGGTATCAAC | GCAGAGTGTTCCAGTCCAGAATCAG |
| *UNG1* | GTTCTCCTTCTCAACGCTGTCCTC | AGAGCCCCAGAGCAAGAAAACAAG |
| *GAPDH* | CGGAGTCAACGGATTTGGTCGTAT | AGCCTTCTCCATGGTGGTGAAGAC |
| *D-Loop* | CTATCACCCTATTAACCACTCA | TTCGCCTGTAATATTGAACGTA |
| *ND1* | CACCCAAGAACAGGGTTTGT | TGGCCATGGGTATGTTGTTAA |
| *COX1* | CTGTTAGTAGTATAGTGATG CCAGCAGCTAGG | GCCATAACCCAATACCAAACGC |
| *COX2* | AATCGAGTAGTACTCCCGATTG | TTCTAGGACGATGGGCATGAAA |
| *Tert* | CGTACTGCGTGCGTCGGTATG | ACGGCTGGAGGTCTGTCAAGG |

**2. Supplementary Figures**

**
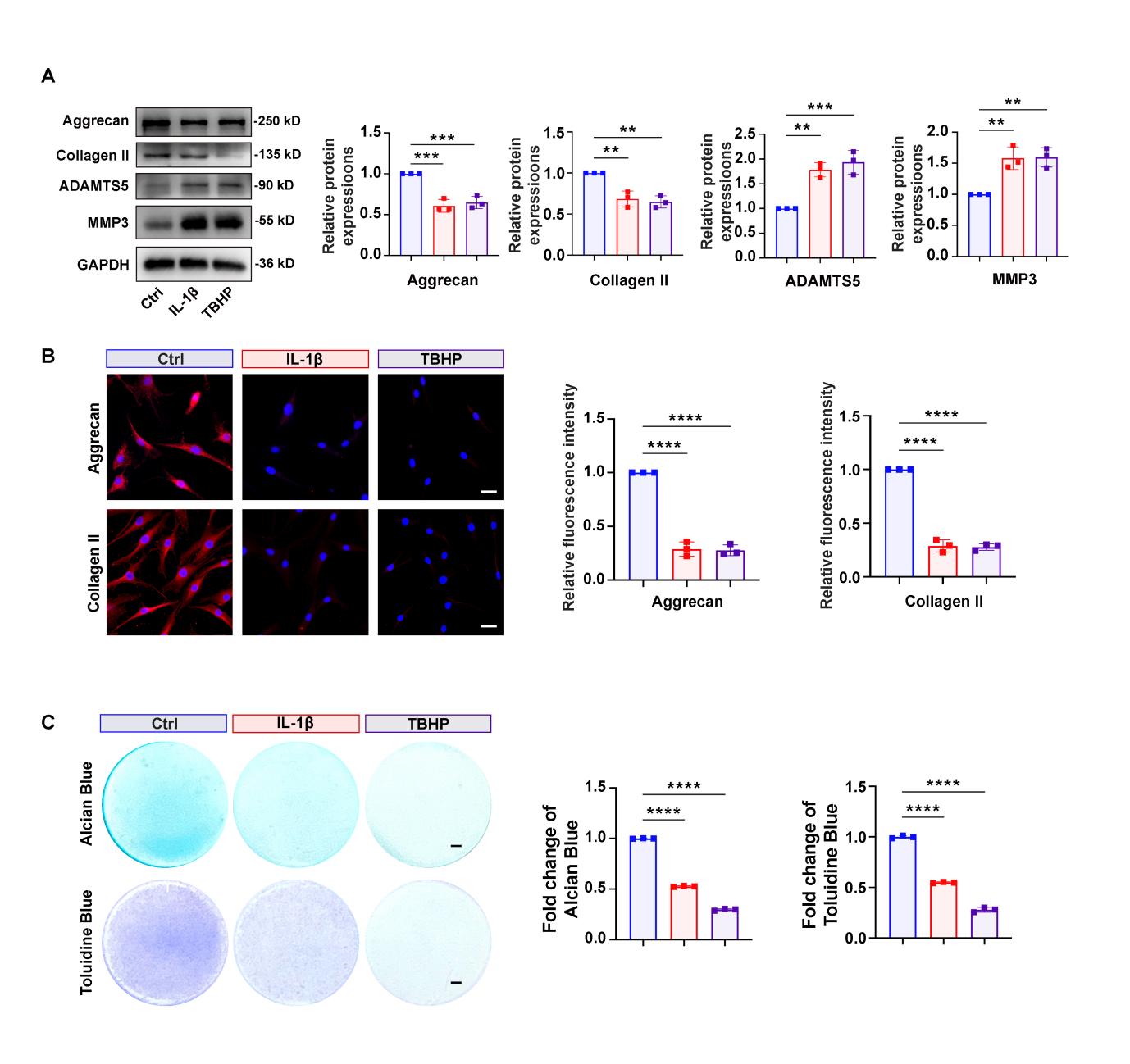
**

**Fig. S1.** (A) Western blotting analysis of Aggrecan, Collagen II, ADAMTS5 and MMP3 expression in IL-1β- or TBHP-treated HCs (n = 3). (B) Representative fluorescence images of Aggrecan and Collagen II in above-treated HCs and its quantification data (scale bar = 25 μm) (n = 3). (C) Alcian blue and toluidine blue staining in above-treated HCs (left) and its quantification data (right) (scale bar = 5 μm) (n = 3). All bar graphs are expressed as the means ± SD. ^**^*P* < 0.01 and ^****^*P* < 0.0001.


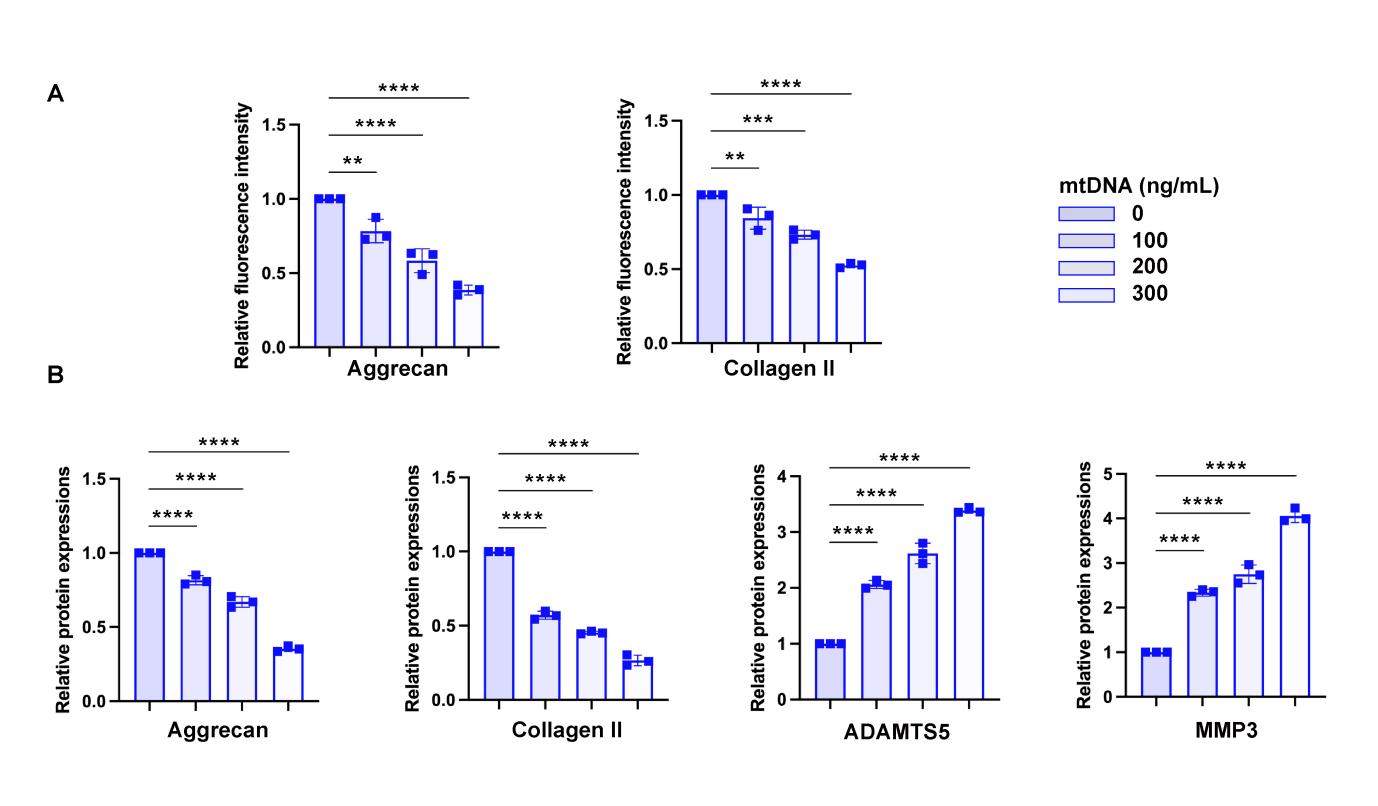


**Fig. S2.** (A) Quantification fluorescence intensity analyses of Aggrecan and Collagen II protein levels in mtDNA-stimulated HCs. (B) Quantification data of Fig. 1H. All bar graphs are expressed as the means ± SD. ^**^*P* < 0.01, ^***^*P* < 0.001, and ^****^*P* < 0.0001.


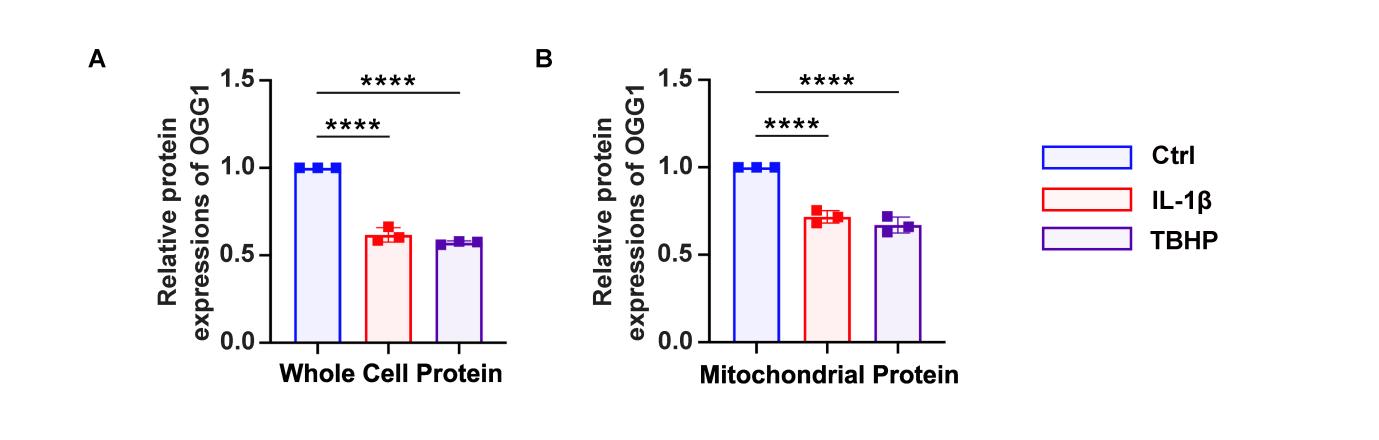


**Fig. S3.** (A-B) Quantification analyses of the whole-cell and mitochondrial OGG1 protein level in stimulated HCs. All bar graphs are expressed as the means ± SD. ^****^*P* < 0.0001.

**
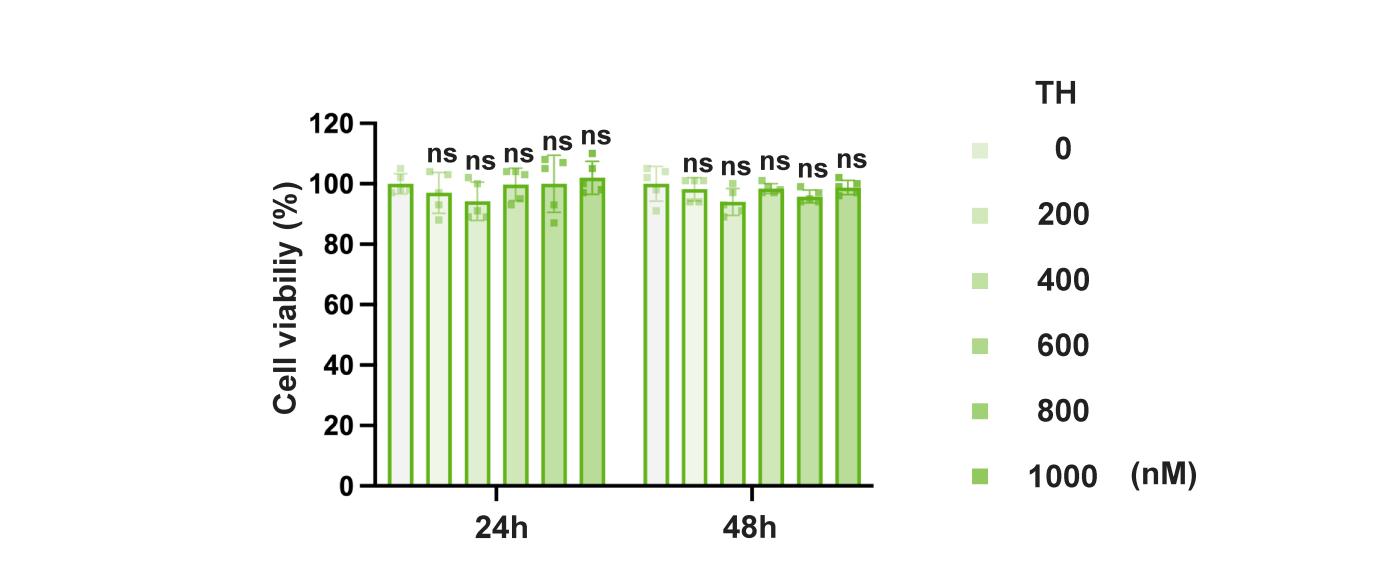
**

**Fig. S4.** TH concentration gradient treatment had no obvious inhibitory effect on the proliferation ability of HCs (n = 5). All bar graphs are expressed as the means ± SD. ^ns^*P* > 0.05.

**
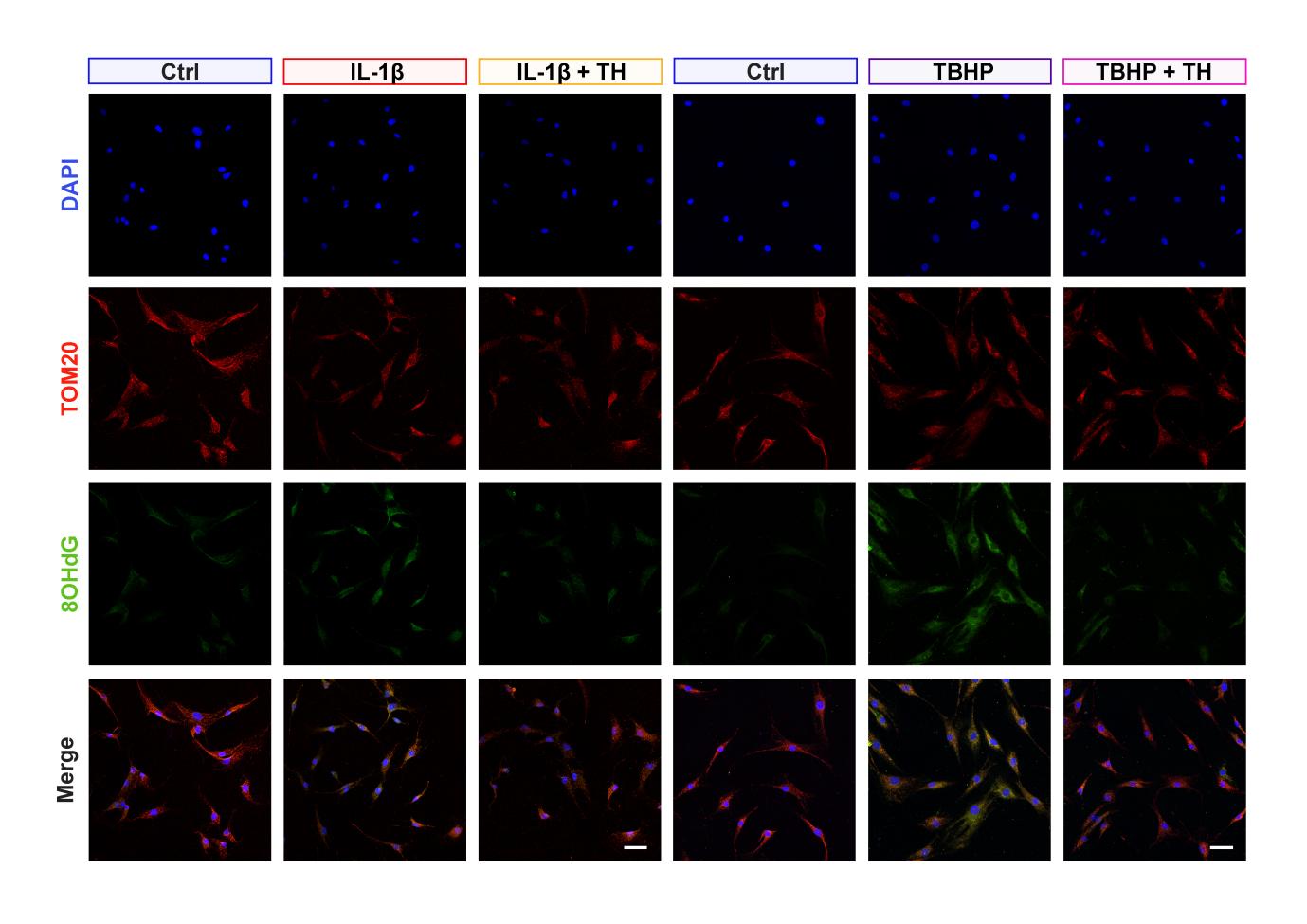
**

**Fig. S5.** Representative fluorescence images of 8OHdG (green), TOM20 (red), and DAPI (blue) in IL-1β- or TBHP-stimulated HCs after TH (1 μM for 24h) treatment (scale bar = 25 μm).


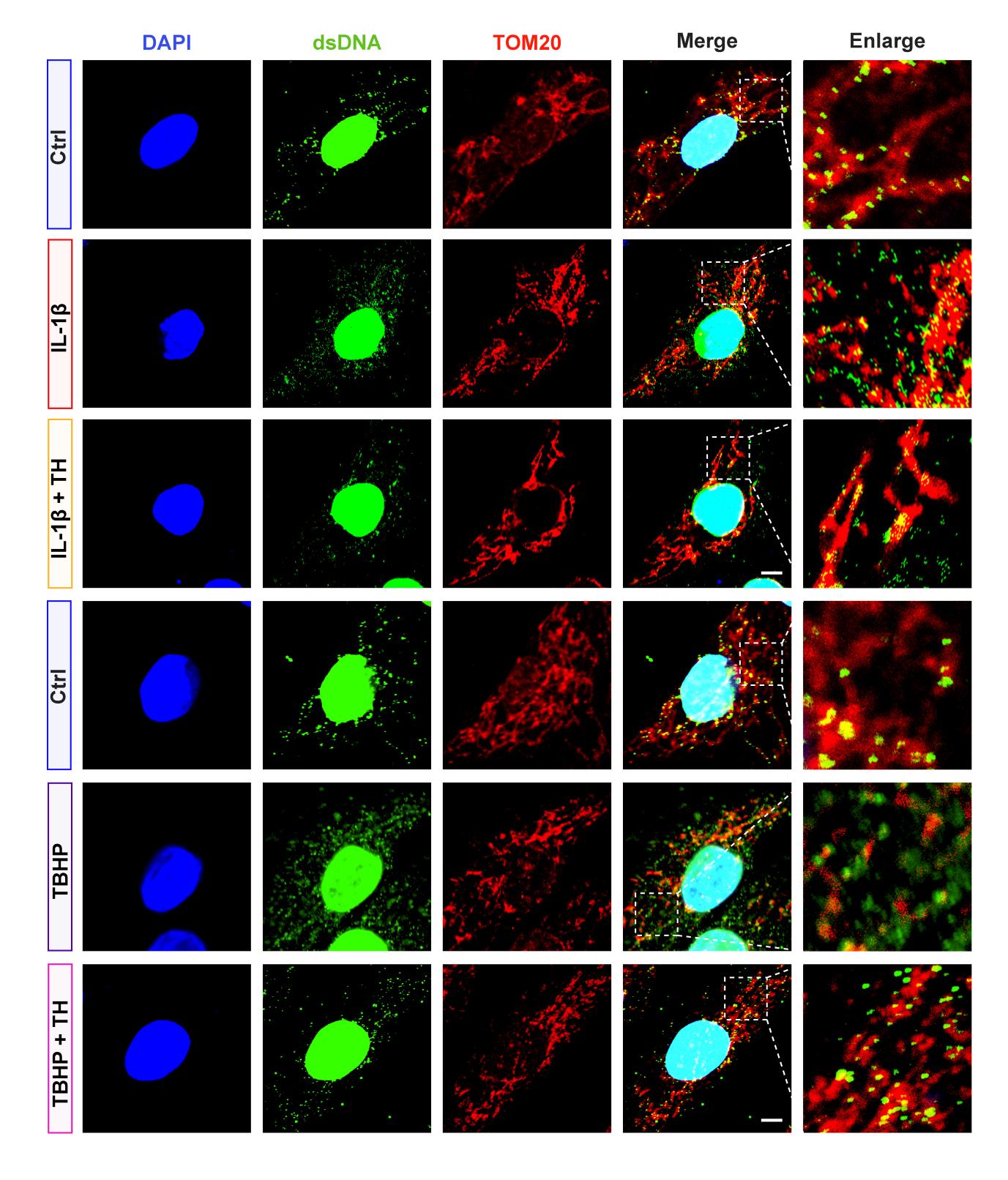


**Fig. S6.** Representative immunofluorescence images of dsDNA (green), TOM20 (red), and DAPI (blue) in above-treated HCs (scale bar = 10 μm).


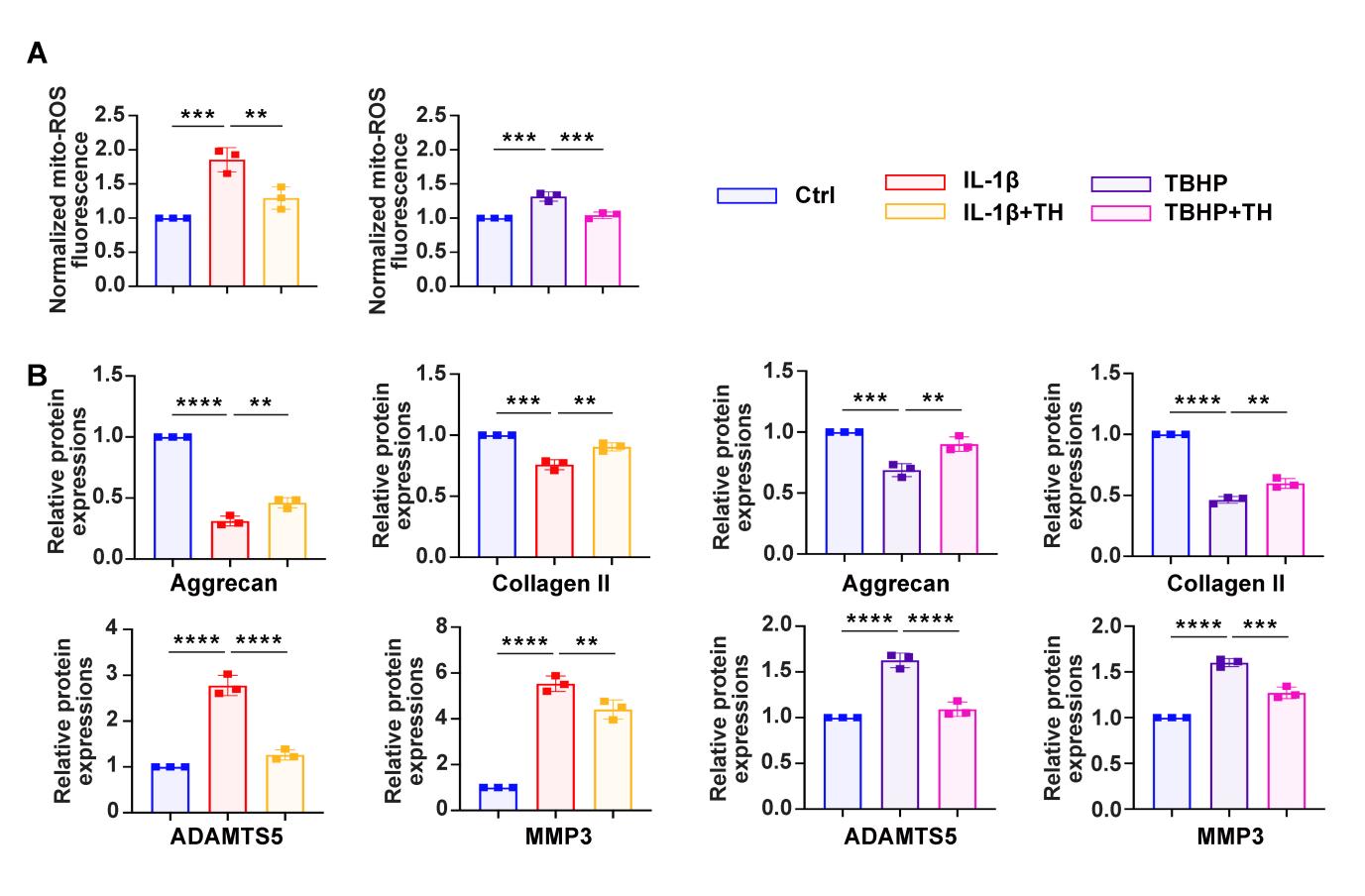


**Fig. S7.** Quantification data of (A) Fig. 2L and (B) Fig. 2M. All bar graphs are expressed as the means ± SD. ^**^*P* < 0.01, ^***^*P* < 0.001, and ^****^*P* < 0.0001.

**
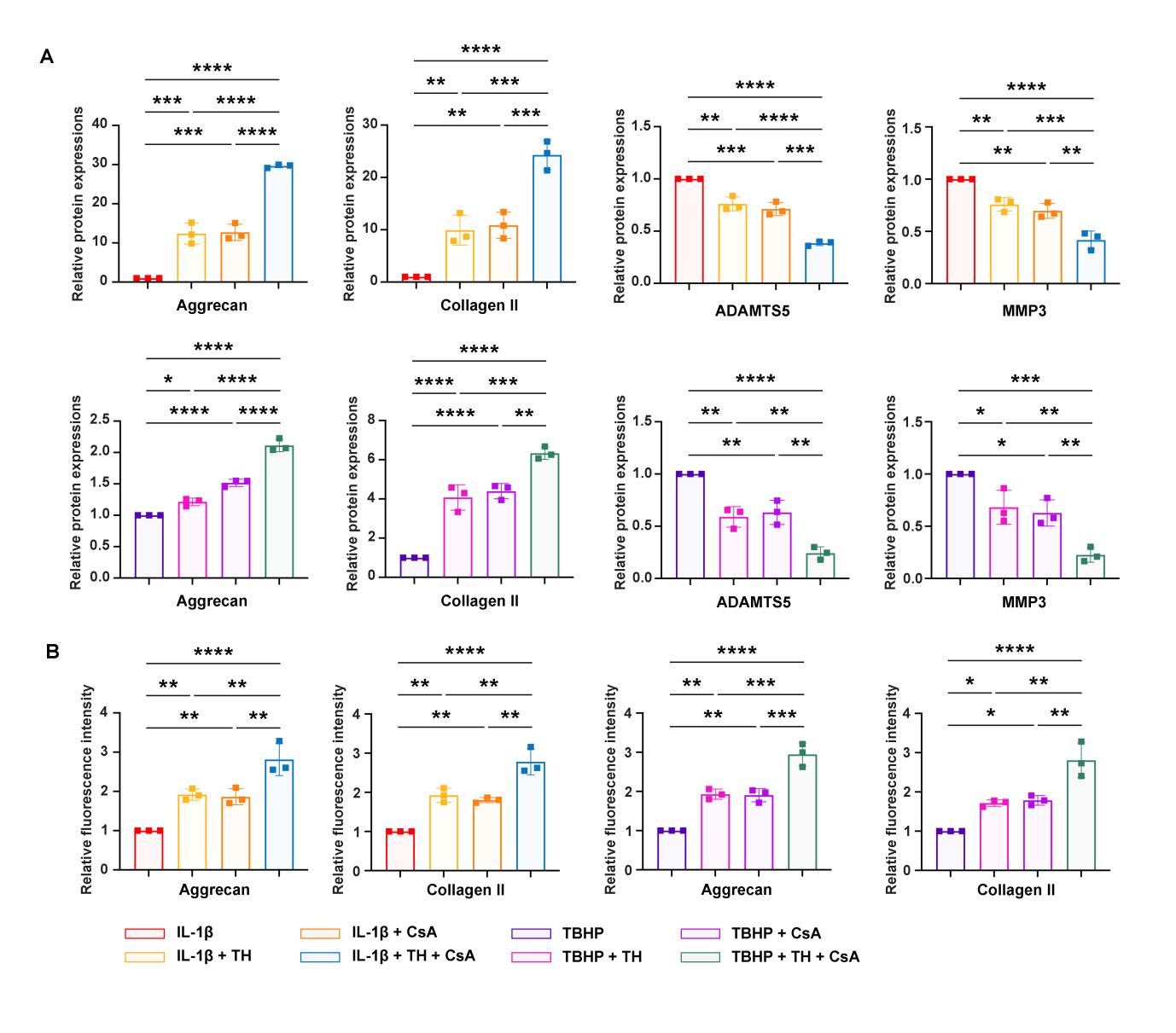
**

**Fig. S8.** (A) Quantification data of Fig. 4E. (B) Quantification data of Fig. 4G. All bar graphs are expressed as the means ± SD. ^*^*P* < 0.05, ^**^*P* < 0.01, ^***^*P* < 0.001, and ^****^*P* < 0.0001.


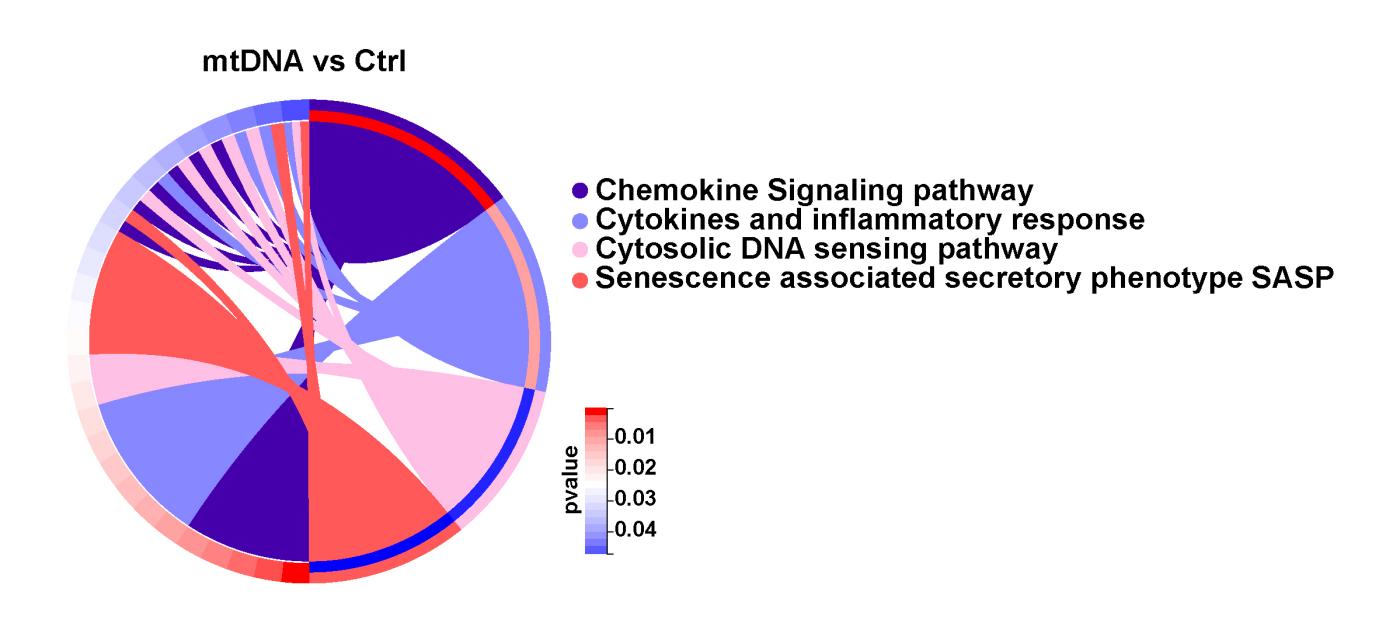


**Fig. S9.** Molecular crosstalk among chemokine signaling pathway, cytokines and inflammatory response, cytosolic DNA sensing pathway and senescence associated secretory phenotype SASP between the mtDNA and control group (*n* = 3).


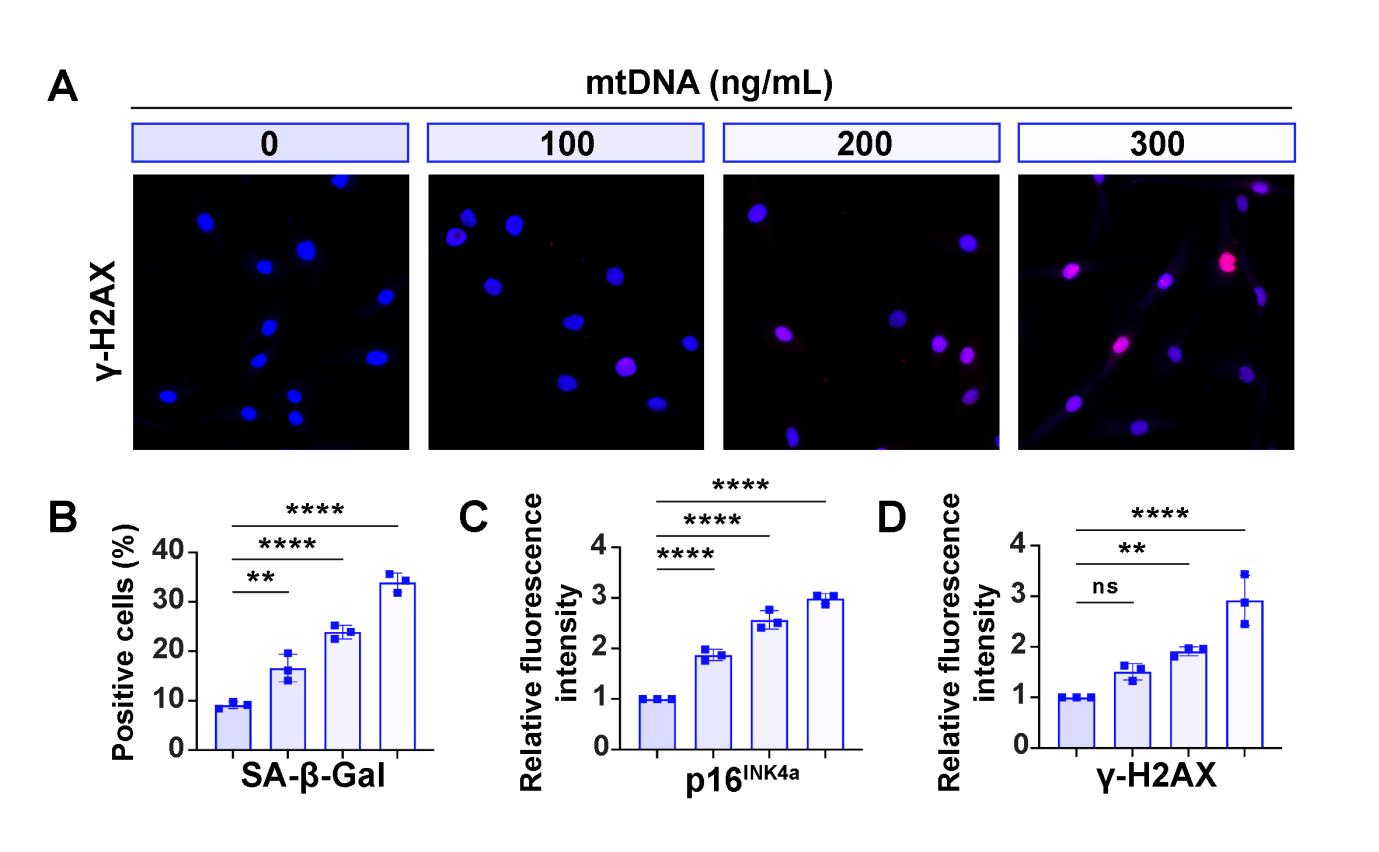


**Fig. S10.** (A) Representative immunofluorescence images of γ-H2AX protein levels in mtDNA-stimulated chondrocytes (n = 3). (B-D) The quantification data of Fig. 5C (scale bar = 25 μm). All bar graphs are expressed as the means ± SD. ^**^*P* < 0.01, ^****^*P* < 0.0001, and ^ns^ > 0.05.


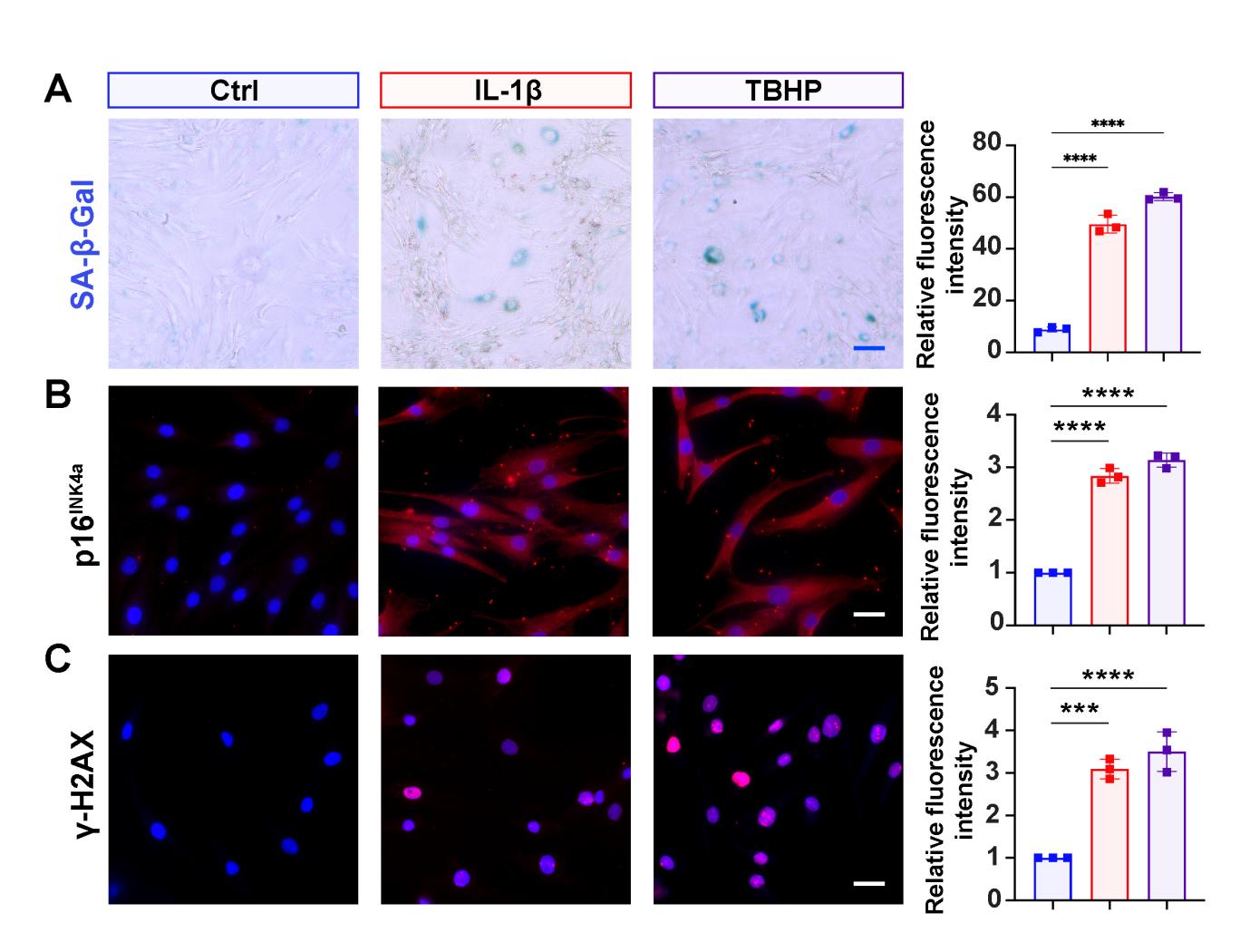


**Fig. S11.** (A) SA-β-gal staining (scale bar = 100 μm) and immunofluorescence of (B) p16^INK4a^/ (C) γ-H2AX (scale bar = 25 μm) in IL-1β- or TBHP-treated HCs (n = 3). All bar graphs are expressed as the means ± SD. ^***^*P* < 0.001, and ^****^*P* < 0.0001.

**
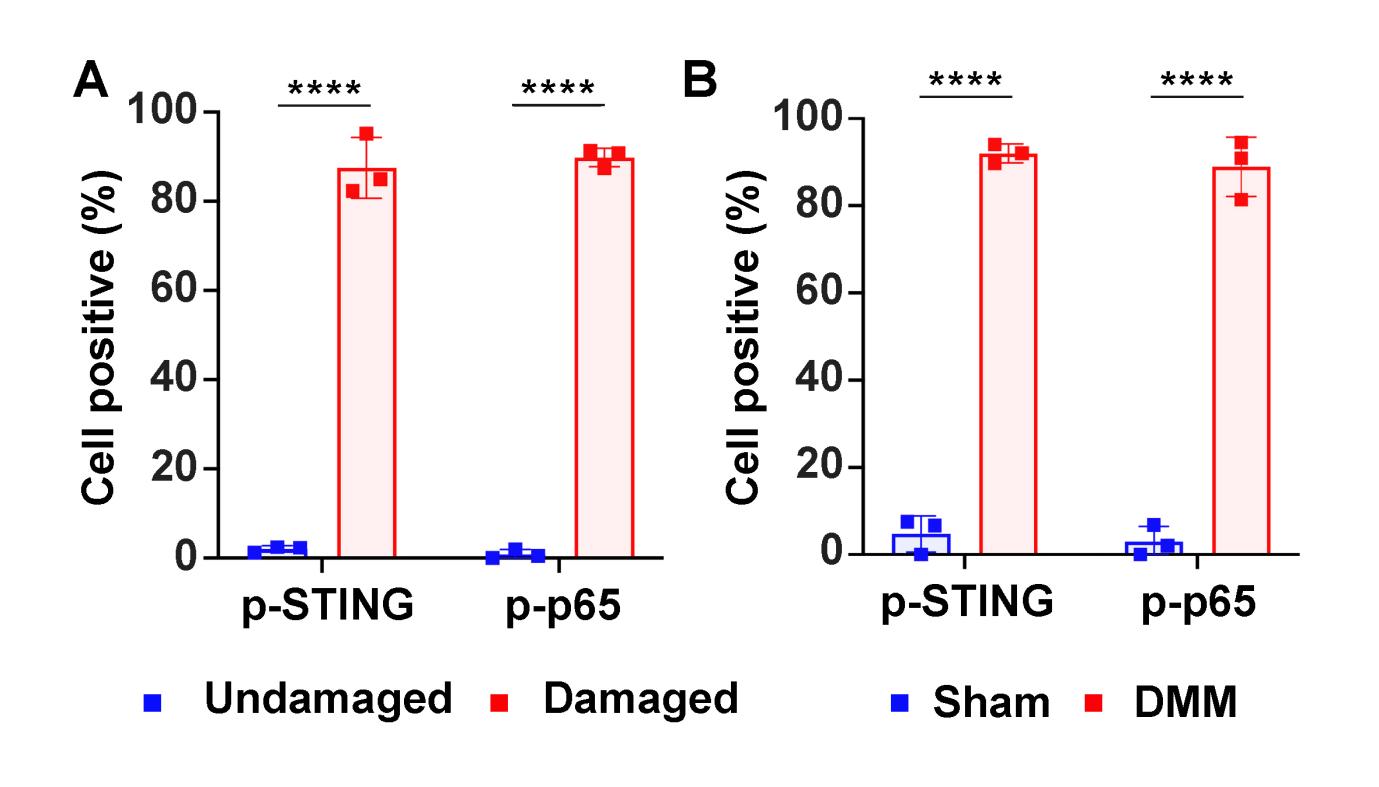
**

**Fig. S12.** The quantification data of p-STING, p-p65 in (A) human and (B) mice OA cartilage (n = 3). All bar graphs are expressed as the means ± SD. ^****^*P* < 0.0001.


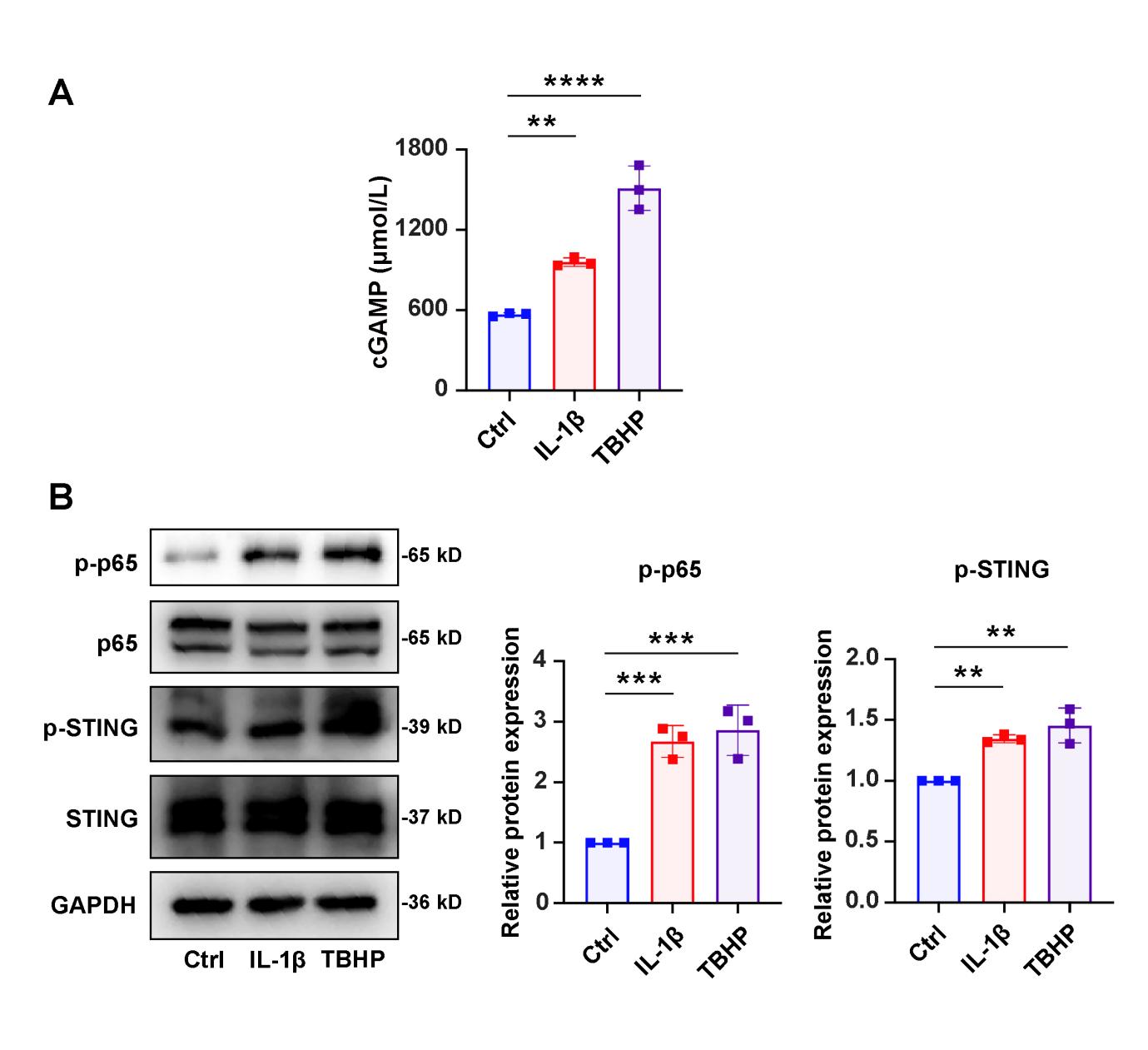


**Fig. S13.** (A) c-GAMP concentrations measured by ELISA in IL-1β- or TBHP-treated HCs (n = 3). (B) Western blotting analysis of p-p65, p65, p-STING and STING expression in IL-1β- or TBHP-treated HCs (n = 3). All bar graphs are expressed as the means ± SD. ^**^*P* < 0.01, ^***^*P* < 0.001, and ^****^*P* < 0.0001.


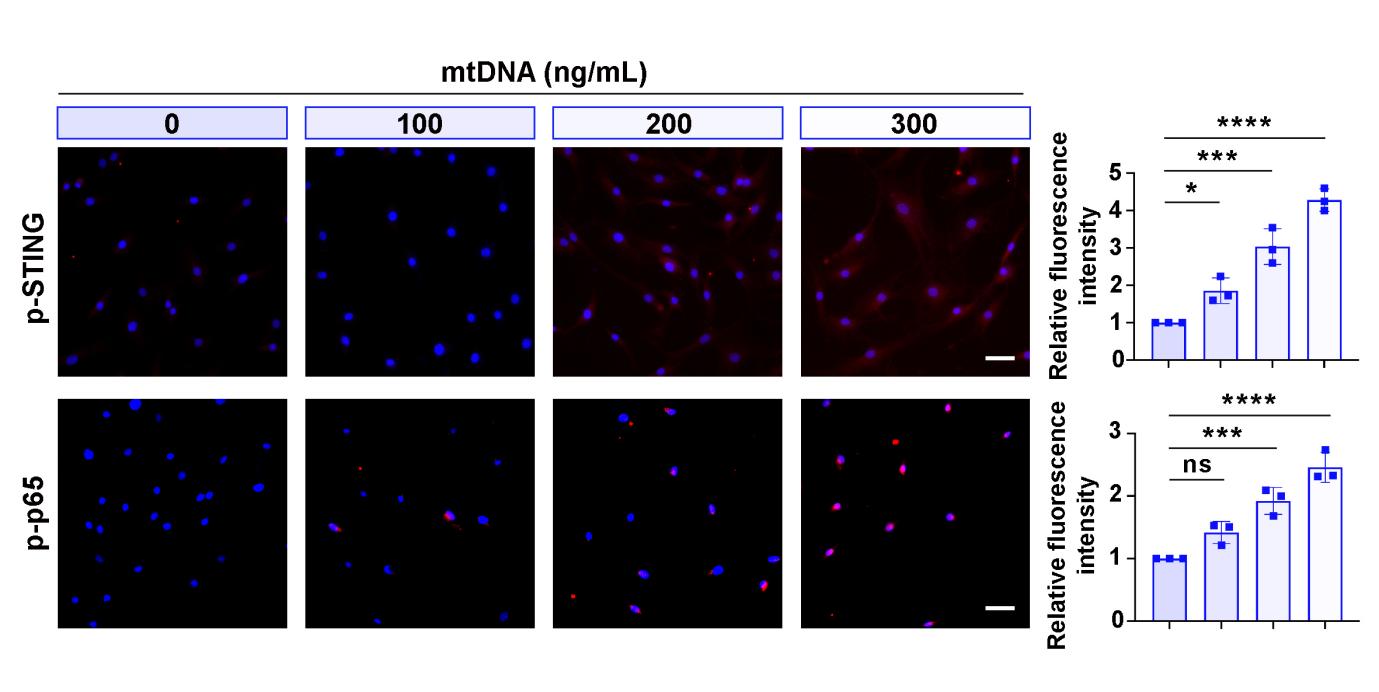


**Fig. S14.** Representative immunofluorescence images of p-p65 and p-STING protein levels in chondrocytes after mtDNA stimulation (scale bar = 25 μm) (n = 3). All bar graphs are expressed as the means ± SD. ^*^*P* < 0.05, ^***^*P* < 0.001, ^****^*P* < 0.0001, and ^ns^ > 0.05.

**
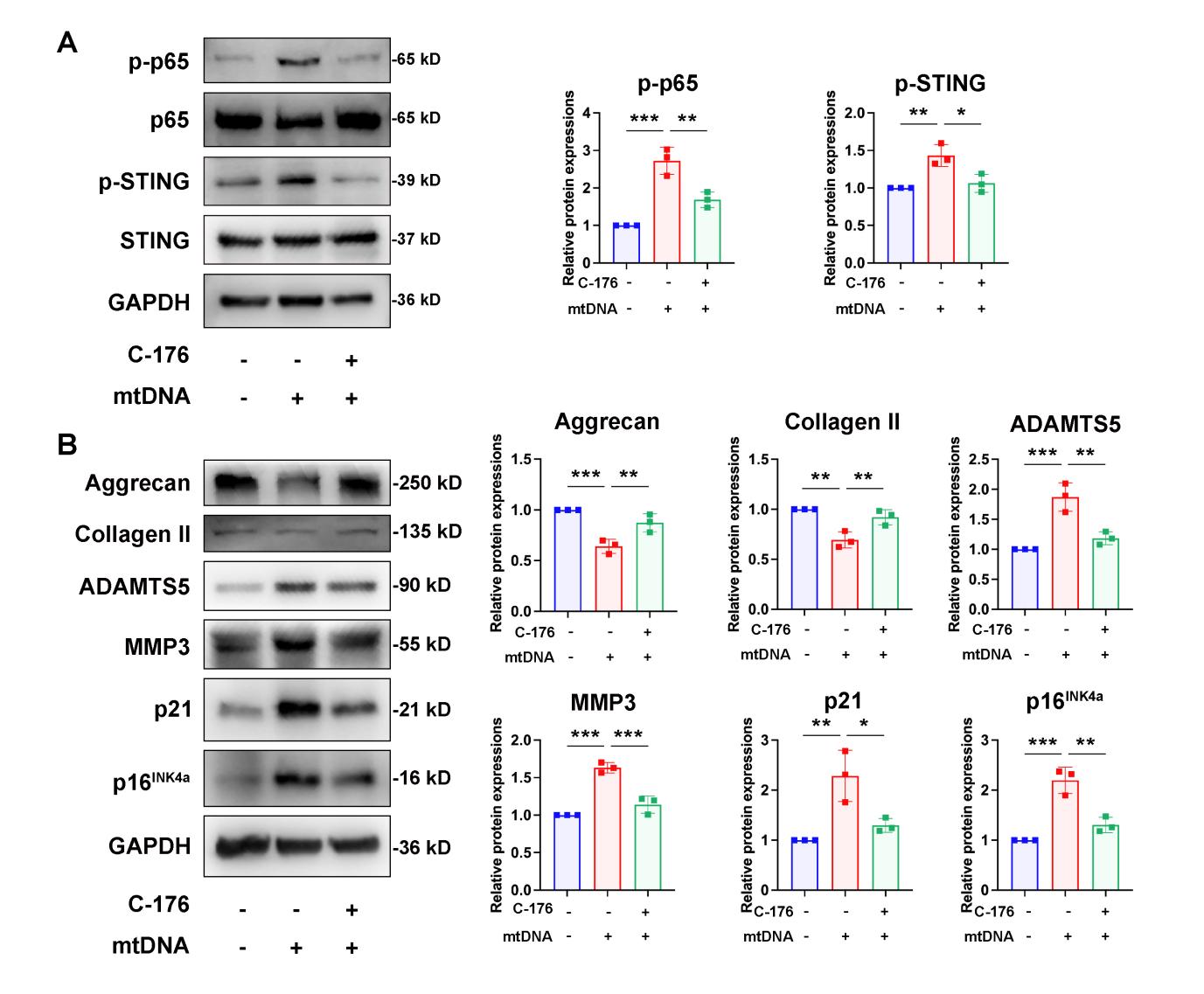
**

**Fig. S15.** (A) Western blotting analysis of p-p65, p65, p-STING and STING expression in mtDNA-treated HCs after C-176 (20 μM for 24h) treatment (n = 3). (B) Western blotting analysis of Aggrecan, Collagen II, ADAMTS5, MMP3, p21 and p16^INK4a^ expression in mtDNA-treated HCs after C-176 treatment (n = 3). All bar graphs are expressed as the means ± SD. ^*^*P* < 0.05, ^**^*P* < 0.01 and ^***^*P* < 0.001.

**
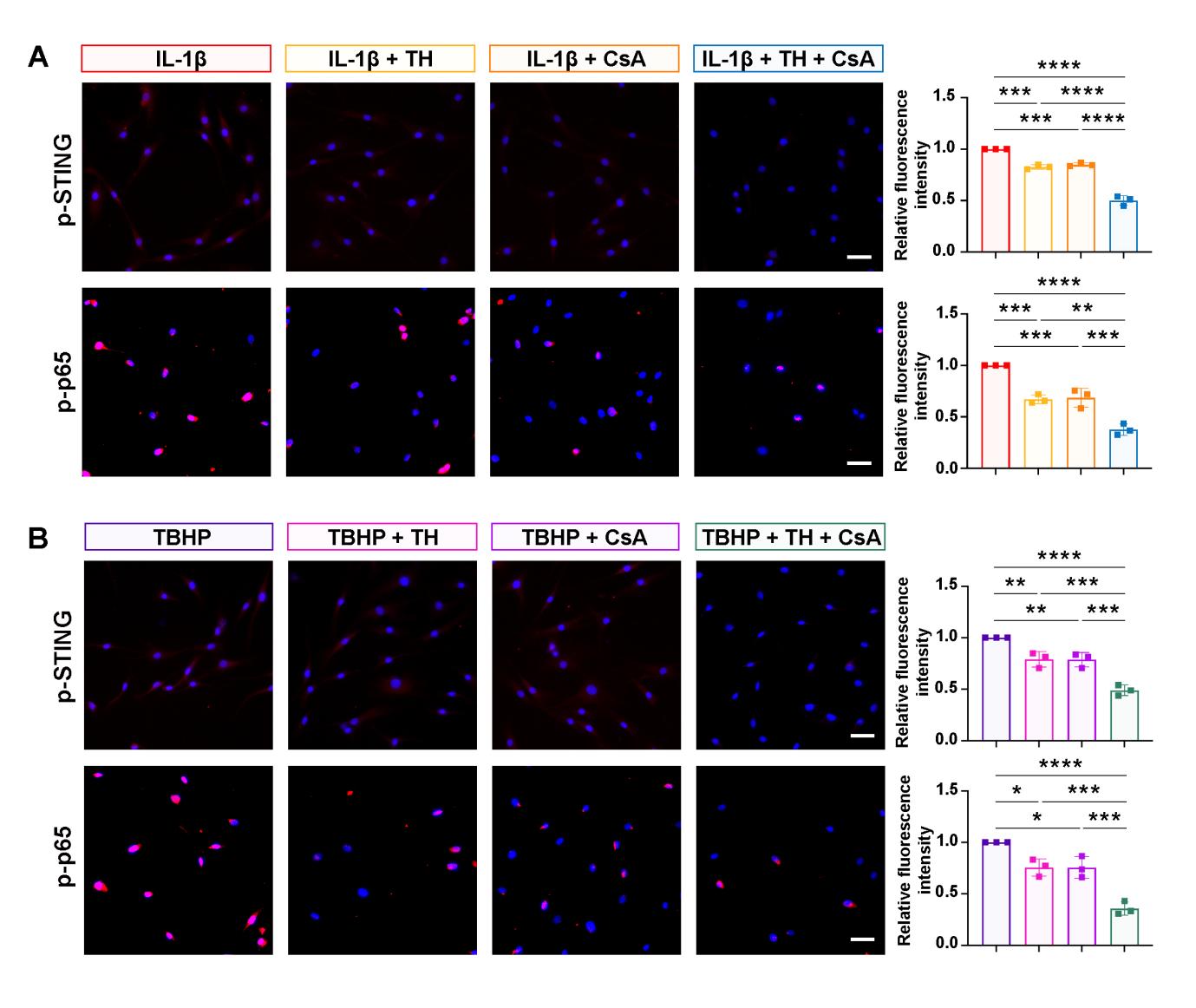
**

**Fig. S16.** Representative immunofluorescence images of p-p65 and p-STING protein levels in IL-1β- (A) or TBHP-stimulated (B) HCs after TH (1 μM for 24h) or/and CsA (1 μM for 24h) treatment (scale bar = 25 μm) (n = 3). All bar graphs are expressed as the means ± SD. ^*^*P* < 0.05, ^**^*P* < 0.01, ^***^*P* < 0.001, and ^****^*P* < 0.0001.


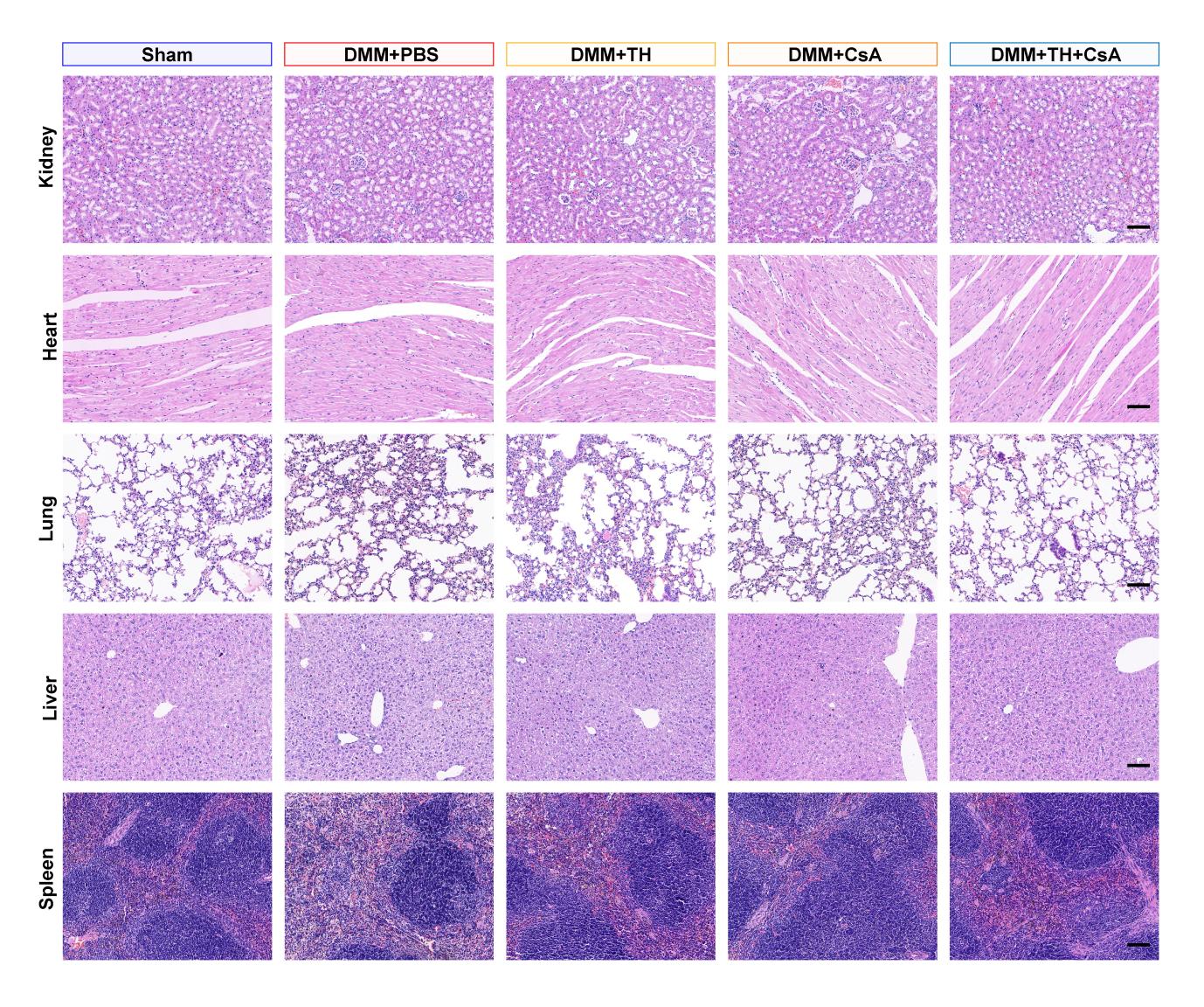


**Fig. S17.** Representative H&E staining images of the kidney, heart, lung, liver and spleen from each group after 8 weeks of treatment (scale bar = 25 μm). No obvious histological impairment was observed in the liver and kidney, and other organs also showed no remarkable pathological abnormalities.
